# The diagnostic value of immune-inflammatory markers for diabetic kidney disease in type 2 diabetic patients: a meta-analysis

**DOI:** 10.3389/fendo.2026.1811189

**Published:** 2026-03-25

**Authors:** Yan Wang, Xiaohua Liu, Zhenwen Xiao

**Affiliations:** 1 Clinical Laboratory Department, Jinan Third People’s Hospital, Jinan, Shandong, China; 2 Department of Nephrology, Jinan Third People’s Hospital, Jinan, Shandong, China

**Keywords:** diabetic nephropathies, early diabetic nephropathy, mean platelet volume, meta-analysis, monocyte-to-lymphocyte ratio, platelet-to-lymphocyte ratio, red blood cell distribution width, systemic immune-inflammation index

## Abstract

**Introduction:**

Diabetic kidney disease (DKD) constitutes a chronic renal condition arising from type 2 diabetes mellitus after excluding other causes. Immune-inflammatory responses are pivotal in the pathogenesis of DKD, and related biomarkers may be diagnostic targets.

**Methods:**

The current meta-analysis appraises the diagnostic value of common immune-inflammatory indicators—red blood cell distribution width (RDW), monocyte-to-lymphocyte ratio (MLR), systemic immune-inflammation index (SII), platelet-to-lymphocyte ratio (PLR), mean platelet volume (MPV), and systemic inflammation response index (SIRI)—for early and established DKD.

**Results:**

We systematically retrieved the Cochrane Library, Embase, PubMed, Web of Science, CNKI, CBM, VIP, and Wanfang databases up to October 11, 2025. The QUADAS-2 tool was applied to evaluate study quality. Meta-analyses were implemented employing Stata 16.0, RevMan 5.3, and MetaDisc 1.4.

**Conclusion:**

Thirty-two eligible investigations were incorporated: 11 on early DKD(649 subjects) and 21 on DKD(9,120 subjects). The meta-analysis yielded pooled diagnostic performance metrics. For early DKD, PLR showed a sensitivity of 0.74 (95% CI: 0.65–0.81), specificity of 0.69(0.54–0.81), and AUC of 0.78(0.74–0.81). For established DKD, SII demonstrated a sensitivity of 0.68(0.59–0.75), specificity of 0.64 (0.54–0.72), and an AUC of 0.71(0.67–0.74). PLR, MLR, MPV, and RDW exhibited low to moderate diagnostic accuracy for both stages (AUC range: 0.68–0.74). Common immune-inflammatory markers have diagnostic value for early and established DKD. Among them, PLR offers moderate diagnostic accuracy for early DKD, while SII performs relatively better for diagnosing DKD. These findings should be verified through future high-quality studies due to limitations of eligible research.

**Systematic review registration:**

https://www.crd.york.ac.uk/prospero/, Identifier CRD420251174942.

## 1 Background

Diabetic kidney disease (DKD) affects up to 40% of people with type 2 diabetes mellitus (T2DM) and 30% with type 1 diabetes mellitus (1). It remains the principal cause of end-stage renal disease (ESRD) and chronic kidney disease worldwide (2). The typical clinical progression of DKD initiates with glomerular hyperfiltration, followed by microalbuminuria (urinary albumin-to-creatinine ratio [UACR]: 30–300 mg/g), macroalbuminuria (UACR > 300 mg/g), and a gradual reduction in estimated glomerular filtration rate (eGFR). These processes culminate in ESRD requiring dialysis (3–5). According to the “DKD: Consensus Report from the American Diabetes Association”, DKD should be diagnosed and monitored based on the evaluation of renal function and injury. Renal function should be evaluated employing eGFR with a cut-off of < 60 mL·min^−1^·(1.73 m²)^−1^, while kidney injury should be determined as UACR ≥ 30 mg/g (6). Renal biopsy remains the diagnostic gold standard but is reserved when superimposed kidney disease is suspected, such as dysmorphic red blood cells, red cell casts, positive glomerulonephritis serology, or atypical proteinuria acceleration (7). It cannot serve as a routine screening tool. Albuminuria has long represented the core clinical feature of DKD. Recent investigations, however, reveal that non-albuminuric DKD is the fastest-growing and most prevalent subtype among individuals with T2DM who experience declined eGFR, and it comprises up to 55% of cases. These individuals present only reduced eGFR without elevated albuminuria, resulting in a significant risk of missed diagnosis for UACR-based screening (8, 9). Given the limited capacity of current diagnostic indices to address the pathological heterogeneity of DKD, developing new biomarkers applicable to both albuminuric and non-albuminuric DKD has become a major research focus.

The pathogenesis of DKD involves complex gene-environment interactions. Glucose metabolism dysregulation, chronic inflammation, and oxidative stress serve as central drivers (10). While cytokines like tumor necrosis factor receptor 1 (TNFR1), TNFR2 (11), interleukin (IL)-6, and transforming growth factor-β1 (10) play crucial regulatory roles in DKD-associated inflammation, these indicators cannot be tested routinely due to high cost. Conversely, novel immune-inflammatory indices derived from routine blood tests offer both economic and practical advantages. These comprise the red blood cell distribution width (RDW), monocyte-to-lymphocyte ratio (MLR), systemic immune-inflammation index (SII), platelet-to-lymphocyte ratio (PLR), mean platelet volume (MPV), neutrophil-to-lymphocyte ratio (NLR), and systemic inflammation response index (SIRI). By reflecting states like neutrophil, lymphocyte infiltration, and platelet activation, these indices are indirectly linked to pathological processes such as glomerular endothelial injury and renal interstitial fibrosis (12, 13), presenting a promising diagnostic direction.

Multiple studies have investigated linkages between inflammatory markers and DKD, yet conclusions exhibit notable heterogeneity. Daniel-Corneliu Leucuta et al.’s meta-analysis (14) indicates a strong link between RDW and DKD risk, proposing the predictive potential of RDW. Their analysis finds no correlation of PLR and SII with DKD, although one longitudinal study confirms SII as a marked predictor for the progression of DKD. A recent meta-analysis by Yijue Wang et al. (13) examines a broader range of markers. They observe notably elevated MLR, PLR, and SII levels in DKD versus non-DKD individuals. Their analysis associates MLR, SII, and SIRI with DKD risk but finds no statistical support for the diagnostic value of PLR. Wenli Liu et al. (15) provide a distinct perspective, demonstrating that high SIRI constitutes an independent risk factor for DKD. Their work further links elevated SII with increased renal disease risk in biopsy-confirmed DKD patients. Conversely, Suyan Duan et al. (16) report conflicting conclusions. Their research associates higher PLR with an elevated risk of deterioration of DKD and validates PLR as an independent risk factor for renal outcomes. Juxiang Liu et al.’s meta-analysis (17) shows higher MPV and RDW levels in DKD patients compared to controls, suggesting MPV as a low-cost, accessible diagnostic marker.

In summary, the correlation of inflammatory markers like PLR, SII, MLR, MPV, RDW, and SIRI with DKD varies across studies. Further investigation into their diagnostic potential holds clinical relevance. Our team previously published a meta-analysis on the diagnostic value of NLR for DKD and early DKD, providing robust evidence for the clinical application of NLR (18). Currently, no meta-analysis systematically evaluates these inflammatory indices or evaluates their stratified diagnostic performance for early DKD and non-albuminuric DKD. This research employs evidence-based medicine and meta-analysis methodologies to systematically incorporate relevant clinical studies to comprehensively appraise the diagnostic efficacy of these inflammatory markers. The aim is to identify reliable options for early DKD screening and disease assessment while furnishing high-quality evidence to support clinical translation.

## 2 Methods

This meta-analysis strictly adhered to the Preferred Reporting Items for Systematic Reviews and Meta-Analyses for Diagnostic Test Accuracy (PRISMA-DTA) guidelines (19). The research protocol has been registered in the International Prospective Register of Systematic Reviews (CRD420251174942).

### 2.1 Literature retrieval

A systematic search was conducted across Chinese databases (China National Knowledge Infrastructure, Wanfang, Chinese Biomedical Literature Database, VIP) and English databases (Cochrane Library, Embase, PubMed, Web of Science) up to October 11, 2025. Search strategies combined Medical Subject Headings and free-text terms. Key terms comprised: (Diabetic Nephropathies OR Diabetic Kidney Disease OR Diabetic Kidney Diseases OR Diabetic Nephropathy) AND (Platelet-to-lymphocyte ratio OR Systemic immune-inflammation index OR Monocyte-to-lymphocyte ratio OR Mean platelet volume OR Red blood cell distribution width OR Systemic inflammation response index). **Supplementary Table 1** provides the details.

### 2.2 Eligibility criteria

#### 2.2.1 Inclusion criteria

i) Population: Adult individuals diagnosed with early DKD or DKD. ii) Diagnostic markers: At least one of PLR, SII, MLR, MPV, RDW, or SIRI. Calculation equations were standardized: PLR = Platelet count/Lymphocyte count, SIRI = (Neutrophil count × Monocyte count)/Lymphocyte count, MLR = Monocyte count/Lymphocyte count, SII = (Platelet count × Neutrophil count)/Lymphocyte count. iii) Diagnostic reference standard: Renal biopsy (gold standard) or clinical criteria including urinary albumin excretion rate ≥ 30 mg/24 h, UACR ≥ 30 mg/g, and/or eGFR < 60 mL·min^−1^·(1.73 m²)^−1^. UACR < 30 mg/g indicated normoalbuminuria, 30–300 mg/g indicated microalbuminuria (early DKD), and > 300 mg/g indicated macroalbuminuria (clinical DKD). iv) Outcome measures: Reported sensitivity and specificity, or data allowing extraction of false positive, true positive, false negative, and true negative values. v) Study type: Observational studies encompassing case-control, cross-sectional, or cohort designs.

#### 2.2.2 Exclusion criteria

i) Non-original research (reviews, case reports, conference abstracts, guidelines, letters, animal experiments). ii) Duplicate publications or unavailable full texts. iii) Investigations with irrelevant biomarkers or diseases. iv) Literature lacking extractable diagnostic outcome data. v) Non-English or Non-Chinese publications.

### 2.3 Literature screening

EndNote 20 was applied to manage acquired records. Two independent researchers (YW and XHL) screened the literature. Duplicates were first removed using software and manual review. Titles and abstracts were then reviewed per the eligibility criteria. Full texts of possibly eligible studies were read to determine final eligibility. Discrepancies were adjudicated by consulting a third researcher (ZWX).

### 2.4 Data extraction

Two researchers (WY and LXH) collected data independently. Collected information encompassed: i) Study characteristics: publication year, first author, country. ii) Participant characteristics: age, sex distribution, sample size, DKD stage or subtype. iii) Reference standard: specific criteria used for DKD diagnosis in each study. iv) Outcome measures: specificity, diagnostic 2×2 table data, sensitivity, area under the summary receiver operating characteristic (SROC) curve (AUC). Discrepancies were addressed by consensus with the third researcher (ZWX).

### 2.5 Quality evaluation

Study quality and applicability were appraised independently by two investigators (WY and LXH) employing the Quality Assessment of Diagnostic Accuracy Studies 2 (QUADAS-2) tool (20). This instrument evaluates risk of bias across four domains (reference standard, patient selection, index test, flow and timing) and concerns regarding applicability in three domains (reference standard, index test, patient selection). A domain was classified as ‘low risk’ only if all items within that domain were answered “yes”. It was rated as ‘high risk’ if any item was answered “no”. It was rated as ‘unclear’ when insufficient information was available to make a definitive judgment. Evaluations were conducted in RevMan 5.3, with disagreements resolved by the third investigator (ZWX).

### 2.6 Statistical analysis

Data on diagnostic accuracy were synthesized using Meta-Disc 1.4 and the MIDAS module in Stata 16.0, employing a bivariate random-effects model. This model accounts for inter-study heterogeneity, threshold effects, and sample size variations while preserving the bivariate nature of original data. Pooled estimates for specificity, sensitivity, positive likelihood ratio (PLR), negative likelihood ratio (NLR), diagnostic odds ratio (DOR), and diagnostic score (DS) were computed and presented in forest plots. Higher DOR and DS values indicated better diagnostic performance. SROC curves were generated, and the AUC was computed. AUCs were interpreted as low (0.5–0.7), moderate (0.7–0.9), or high (0.9–1.0) diagnostic accuracy. Sensitivity analysis was implemented to evaluate the robustness of the results. Threshold effects were detected via the Spearman’s correlation coefficient (*P* > 0.05 suggested an absence of heterogeneity). Cochran’s Q test and the I² statistic were applied to quantify heterogeneity. A random-effects model was applied when *P* < 0.10 or *I²* > 50%; otherwise, a fixed-effects model was applied. For markers exhibiting high heterogeneity, possible sources were detected via subgroup analyses and meta-regression (e.g., DKD subtype, diagnostic method, region). Publication bias was examined via Deeks’ funnel plot, with *P* < 0.05 indicating potential bias.

## 3 Results

### 3.1 Literature screening

**Figure 1:**
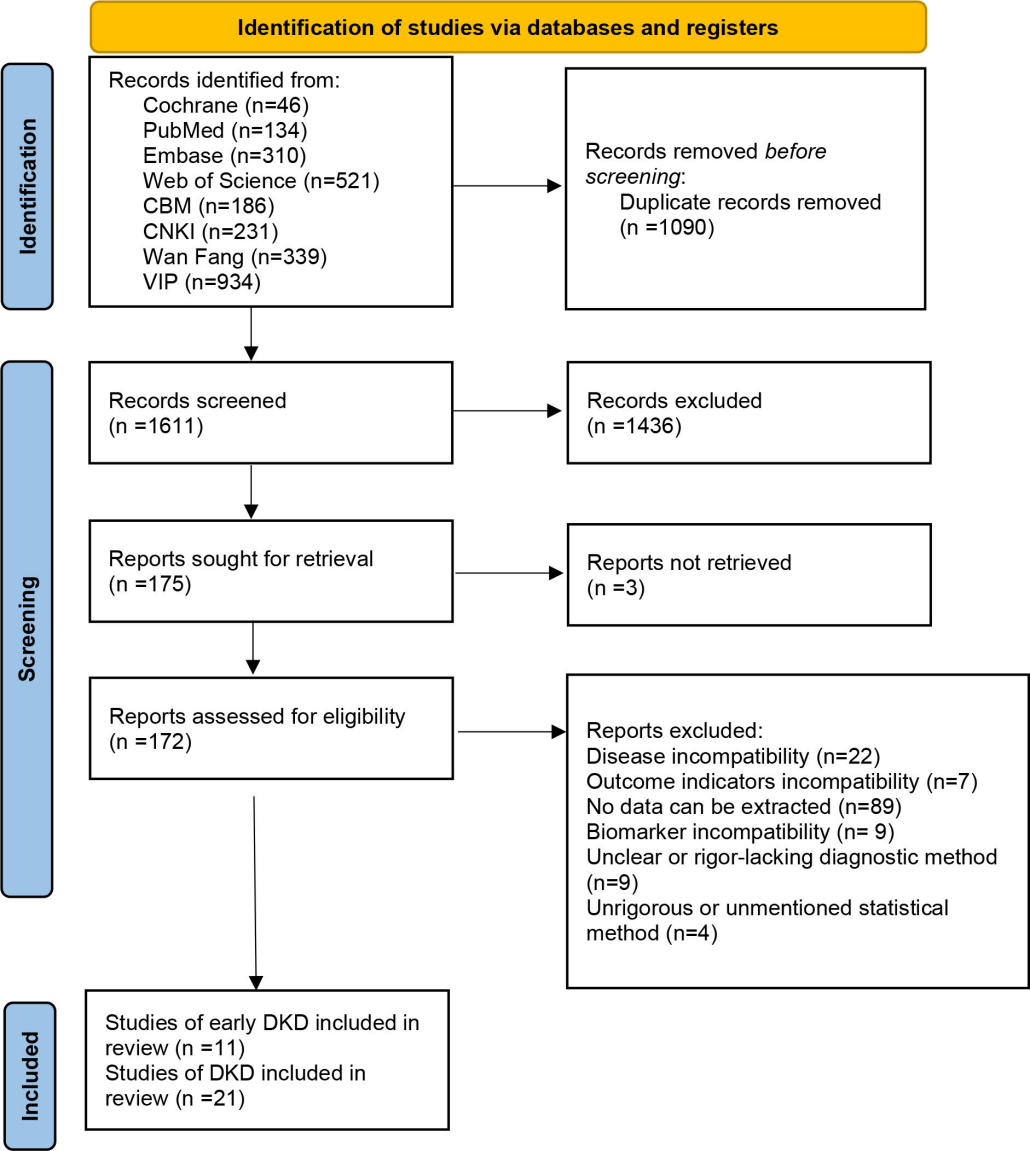
PRISMA flow diagram of literature screening.

The initial database search yielded 2,701 records. After removing 1,090 duplicates, 1,611 titles and abstracts were screened, and 1,436 records were removed. Full texts of the remaining 175 articles were read, and 143 articles were removed due to irrelevant disease (n = 22), irrelevant outcomes (n = 7), insufficient data (n = 89), irrelevant biomarker (n = 9), ambiguous diagnostic methods (n = 9), or inadequate statistical reporting (n = 4). Finally, 32 studies met all inclusion criteria: 11 focused on early DKD (21–31) and 21 on DKD (32–52). The literature screening process is displayed in **Figure 1**.

### 3.2 Study characteristics

The 32 eligible studies (21–52) evaluated six inflammatory markers: PLR, SII, MLR, MPV, RDW, and SIRI. The 11 studies on early DKD (21–31) encompassed PLR (5 studies), MLR (3), MPV (3), RDW (3), and SIRI (1). Ten studies originated from Asia (21–23, 25–31) and one from Africa (24). Participants’ mean age ranged from 46.6 to 64.0 years. Reported cut-offs for PLR ranged from 68.86 to 115.60 (ratio). The 21 studies on DKD (32–52) involved SII (8 studies), PLR (9), MLR (3), MPV (3), RDW (2), and SIRI (2). Nineteen studies were from Asia (32–36, 38, 40–52), one from Africa (37), and one from North America (39). Mean age ranged from 48.9 to 67.9 years. The cut-offs for SII ranged from 327.83 to 624.35 (×10^9^/L), and cut-offs for PLR from 103.28 to 137.56 (ratio). **Table 1** presents key study characteristics of the included studies.

**Table 1:** Characteristics of studies.

Study [year]	Region	Study type	Sample size		Age (Mean ± SD)	Gender	DKD subtype	Detected biomarker(s)	Biomarker cut-off value	Diagnostic basis
			Case control			(F/M)				
Xu Chen et al, 2022 (21)	China	CCS	49	134	54.76 ± 11.56	70/113	Early DKD	PLR	68.86	UACR
Min Zhang et al, 2015 (22)	China	CSS	118	202	46.60 ± 7.76	126/194	Early DKD	RDW	12.80	UACR
Susmitha Chollangi et al, 2023 (23)	Bhubaneswar	CSS	45	45	62.25 ± 9.70	37/53	Early DKD	RDW	15.05	UACR
Amira M Mattared et al, 2019 (24)	Egypt	CCS	30	50	59.35 ± 9.87	_	Early DKD	MPV	_	24h-UAER
Mehmet Zahid Kocak et al, 2018 (25)	Turkey	CSS	76	86	58.90 ± 9.58	_	Early DKD	PLR+MPV	PLR:112 MPV:7.73	UACR
Mehmet Zahid Kocak et al, 2020 (26)	Turkey	CC	72	140	59.90 ± 8.54	117/95	Early DKD	MLR	0.22	UACR
Marwa Jaaban et al, 2021 (27)	Syria	CCS	50	67	57.08 ± 8.65	66/92	Early DKD	PLR	115.60	UACR
Tikva Assulyn et al, 2020 (28)	Israel	CCS	58	110	64.02 ± 10.56	84/84	Early DKD	RDW	14.44	UACR+ 24h-UAER
Li Liu et al, 2023 (29)	China	CCS	74	275	59.35 ± 11.43	203/215	Early DKD	MLR+SIRI	_	UACR+ eGFR
Ran ran Huang et al, 2019 (30)	China	CCS	120	241	56.04 ± 10.35	_	Early DKD	PLR+MPV	PLR:106.49 MPV:10.72	UACR
Xiu qin Liu et al, 2022 (31)	China	CCS	65	167	54.40 ± 8.57	96/136	Early DKD	PLR+MLR	PLR:100.20 MLR:5.58	UACR
Pijun Yan et al, 2024 (32)	China	CSS	1063	859	60.72 ± 11.30	947/975	DKD	SII	609.85	UACR+ eGFR
Shuwu Wei 2024 (33)	China	CSS	1481	2821	59.69 ± 14.75	1901/2401	DKD	MPV	_	UACR+ eGFR
Emin Murat Akbas 2014 (34)	Turkey	CSS	68	132	57.28 ± 10.64	103/97	DKD	PLR	135.20	UACR
Lan Li 2022 (35)	China	CSS	365	290	59.94 ± 10.63	304/351	DKD	PLR	125.04	UACR
Tuba Taslamacioglu Duman 2023 (36)	Turkey	CCS	126	413	56.51 ± 12.90	242/297	DKD	SII	336.00	_
Heba Mahmound Mohamed Ibrahim 2024 (37)	Egypt	CCS	60	30	50.25 ± 7.85	51/39	DKD	MLR	0.34	UACR
Qinghua Huang 202038 (38)	China	CCS	99	303	58.23 ± 12.04	141/261	DKD	MLR	0.23	eGFR
Xiaowan Li 2023 (39)	US	CSS	2271	4882	48.91 ± 18.23	3959/3194	DKD	MLR+PLR+SII+SIRI	MLR: 0.22 PLR: 109.18 SII: 327.83 SIRI: 0.72	UACR+ eGFR
A KIYKIM K 2014 (40)	Turkey	CCS	196	367	49.38 ± 13.54	292/281	DKD	RDW	11.80	24h-UAER
Jiaqi Chen 2024 (41)	China	CSS	462	950	63.08 ± 11.59	663/749	DKD	RDW+PLR	_	UACR+ eGFR
Xiaohong Zhang 2024 (42)	China	CCS	108	92	56.60 ± 13.40	72/128	DKD	SII	624.35	Kidney biopsy
Binish Sreekumar 2024 (43)	Kerala	CSS	42	58	54.60 ± 8.90	43/57	DKD	MPV	10.60	UACR
Zhi Shang 2025 (44)	China	CS	1495	8042	62.00 ± 13.00	3702/5835	DKD	SII+SIRI	SII: 576.29 SIRI: 0.93	eGFR
Lizhen Zhao 2023 (45)	China	CCS	215	112	71.20 ± 5.90	155/172	DKD	PLR+SII	PLR: 112.81 SII: 492.08	UACR
Huifang Li2024 (46)	China	CCS	412	661	63.39 ± 10.72	511/562	DKD	SII	_	UACR
Jingyang Li2023 (47)	China	CCS	82	82	67.86 ± 6.45	70/94	DKD	PLR	103.28	UACR+ eGFR
Zhifang Jiang2025 (48)	China	CCS	53	259	55.56 ± 6.12	150/162	DKD	SII	400.00	UACR+ eGFR
Xiaohui Sun 2023 (49)	China	CCS	125	32	52.18 ± 10.72	41/116	DKD	PLR	125.30	Kidney biopsy
Dengyao Liang2024 (50)	China	CCS	200	100	54.11 ± 14.32	_	DKD	PLR	126.14	UACR
Zhezheng Wang2025 (51)	China	CCS	55	55	63.92 ± 11.47	51/62	DKD	PLR	137.56	UACR+ eGFR
Shuqing Zhang2021 (52)	China	CCS	142	243	60.66 ± 13.40	181/204	DKD	MPV	_	24h-UAER

DKD, Diabetic kidney disease; UACR, urinary albumin-to-creatinine ratio; 24h-UAER, 24h-urinary albumin excretion rate; eGFR, estimated glomerular filtration rate; CCS, Case-control study; CSS, Cross-sectional study; CS, Cohort study; _: Not available

### 3.3 Quality evaluation

**Figure 2:**
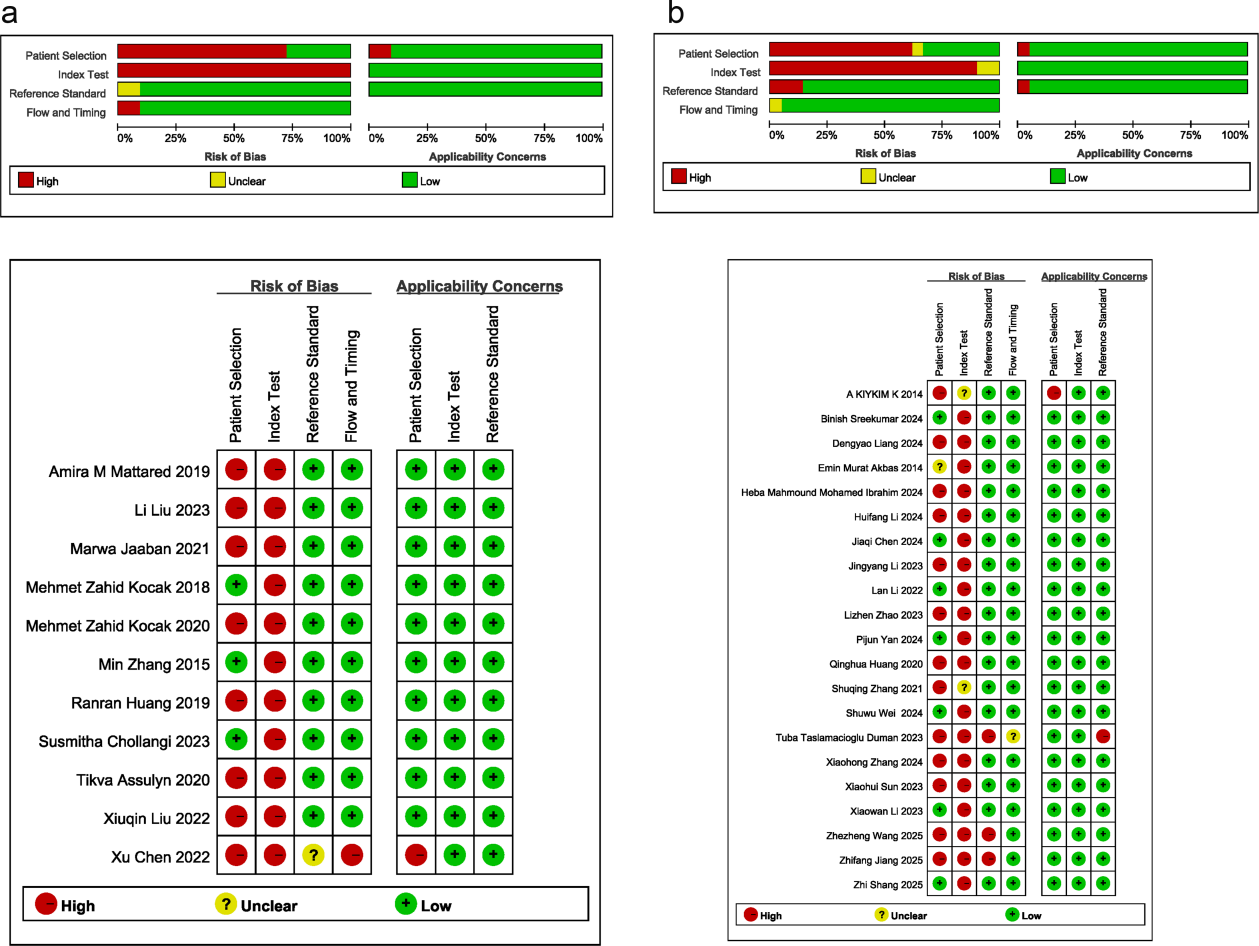
Literature quality evaluation. **(a)** Early DKD studies, **(b)** DKD studies.

Quality of the eligible studies was evaluated utilizing QUADAS-2 in RevMan 5.3. Subgroup-specific quality evaluation revealed a certain risk of bias for studies on both early DKD and DKD. Risk was primarily concentrated in the ‘index test’ and ‘patient selection’ domains. For patient selection, eight studies on early DKD and 13 on DKD were rated as high risk due to case-control design limitations. In the index test domain, 11 studies on early DKD and 19 on DKD were of high risk because the interpretation of the results was not blinded. These methodological limitations may affect the reliability of meta-analysis results. Literature quality evaluation is detailed in **Figure 2**.

### 3.4 Meta-analysis results

#### 3.4.1 Inflammatory markers for early DKD diagnosis

**Figure 3:**
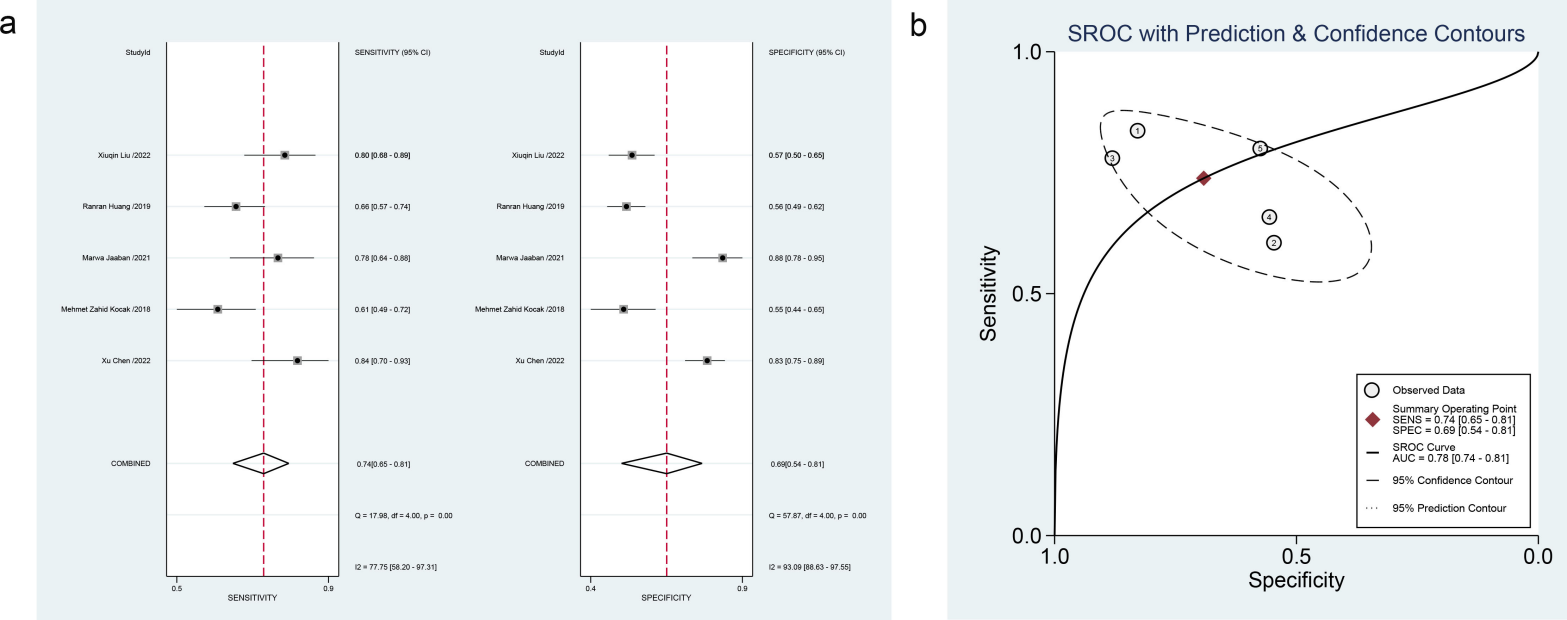
**(a)** Forest plot of PLR for early DKD diagnosis, **(b)** SROC curve of PLR for early DKD diagnosis.

Eleven studies on early DKD (21–31) covered PLR (5 studies), MLR (3 studies), MPV (3 studies), RDW (3 studies), and SIRI (1 study). For PLR, pooled estimates for diagnostic performance were as follows: sensitivity 0.74 (95% confidence interval [CI]: 0.65-0.81, *I²* = 77.75%); specificity 0.69 (0.54-0.81, *I²* = 93.09%); PLR 2.4 (1.4-4.1); NLR 0.38 (0.24-0.61); DOR 6 (2-17); and SROC-AUC 0.78 (0.74-0.81). Forest plots and SROC curves are presented in **Figures 3a, b**. Spearman’s correlation coefficient was -0.700 (*P* = 0.188), indicating no threshold effect. Among other markers, MLR and RDW demonstrated moderate diagnostic value for early DKD, while MPV showed lower value. Details are available in **Table 2**.

**Table 2:** Summary of diagnostic value of other inflammatory markers for early diabetic kidney disease.

Markers	Number of studies (n)	Sensitivity	Specificity	PLR	NLR	OR	ROC-AUC	Spearman coefficient	*P*
MLR	3	0.69	0.65	1.95	0.49	4.01	0.74	0.500	0.667
MPV	3	0.70	0.64	1.94	0.47	4.14	0.68	-0.500	0.667
RDW	3	0.60	0.71	2.08	0.58	3.63	0.74	0.500	0.667

Given evident heterogeneity in the diagnostic power of PLR for early DKD, subgroup analyses were implemented to explore possible sources across four domains: studies conducted in China or not, studies in Asian populations or not, controls limited to T2DM patients or not, and study design (cross-sectional vs. case-control). Only study design notably influenced heterogeneity in sensitivity (*P* = 0.01). The single cross-sectional study reported a pooled sensitivity of 0.61 (95% CI: 0.44-0.77), while four case-control studies showed a higher pooled sensitivity of 0.76 (0.69-0.83). Details of subgroup analyses are available in **Table 3**.

**Table 3:** Subgroup analysis of heterogeneity sources for PLR in diagnosing early diabetic kidney disease.

Subgroup factors		Number of studies (n)	Sen.	P	Spe.	P
Study type	Cross-sectional study	1	0.61 [0.44 - 0.77]	**0.01**	0.55 [0.22-0.87]	0.27
	Case-control study	4	0.76 [0.69 -0.83]		0.72 [0.59 - 0.86]	

Bold value was considered statistically significant.

#### 3.4.2 Inflammatory markers for diagnosis of DKD

**Figure 4:**
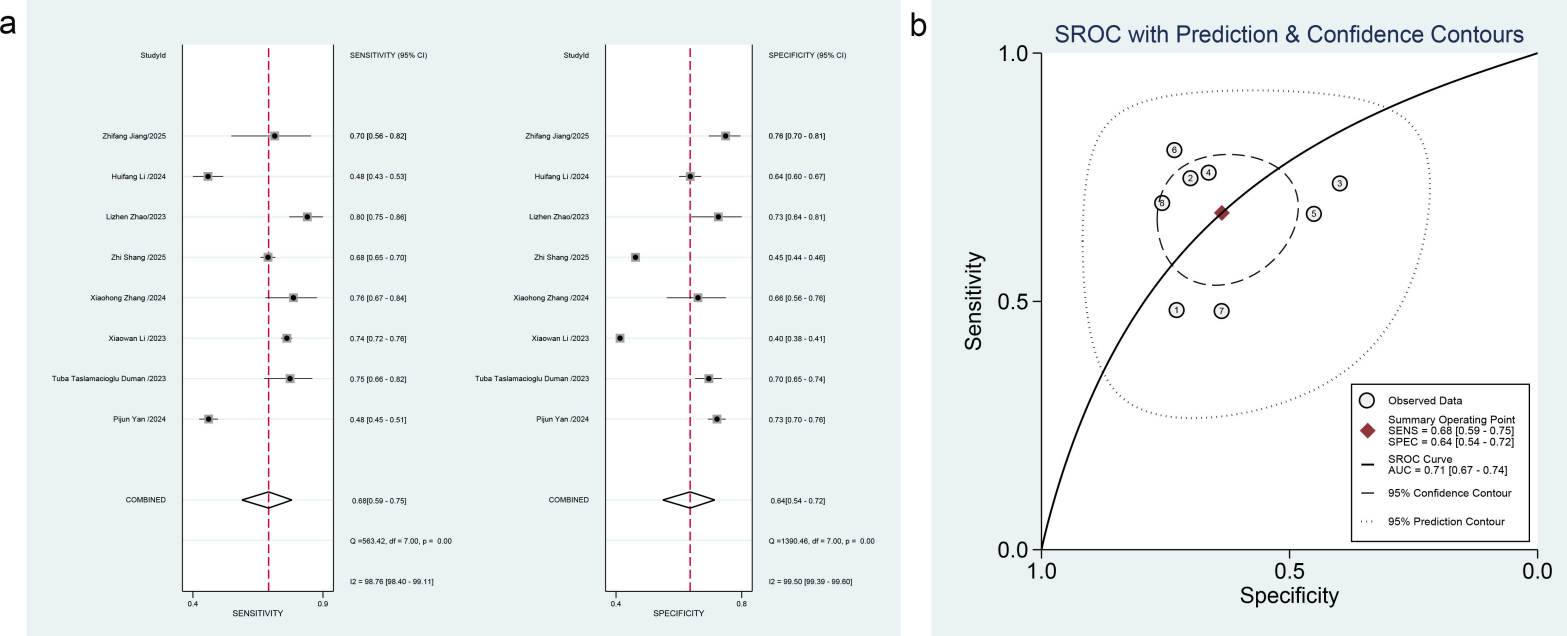
**(a)** Forest plot of SII for DKD diagnosis, **(b)** SROC curve of SII for DKD diagnosis.

Twenty-one studies (32–52) evaluated markers for DKD, including SII (8 studies), PLR (9 studies), MLR (3 studies), MPV (3 studies), RDW (2 studies), and SIRI (2 studies). For SII, pooled estimates were: sensitivity 0.68 (95% CI: 0.59-0.75, *I²* = 98.76%); specificity 0.64 (0.54-0.72, *I²* = 99.50%); PLR 1.9 (1.4-2.4); NLR 0.51 (0.38-0.67); DOR 4 (2-6); SROC-AUC 0.71 (0.67-0.74). Forest plots and SROC curves are shown in **Figures 4a, b**. Spearman’s coefficient was -0.238 (*P* = 0.570), indicating no threshold effect.

**Figure 5:**
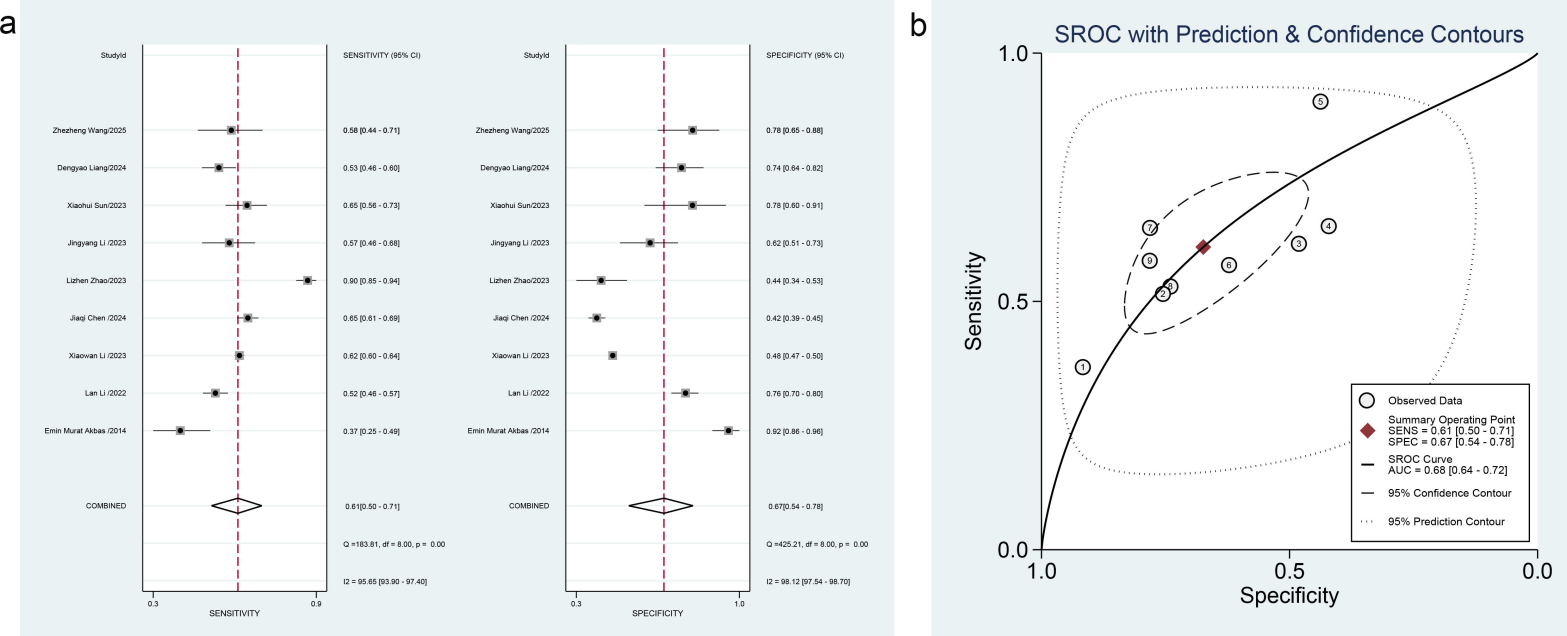
**(a)** Forest plot of PLR for DKD diagnosis, **(b)** SROC curve of PLR for DKD diagnosis.

For PLR, pooled results were: sensitivity 0.61 (95% CI: 0.50-0.71, *I²* = 95.65%); specificity 0.67 (0.54-0.78, *I²* = 98.12%); PLR 1.9 (1.4-2.5); NLR 0.58 (0.47-0.71); DOR 3 (2-5); SROC-AUC 0.68 (0.64-0.72). Forest plots and SROC curves are displayed in **Figures 5a, b**. Spearman’s coefficient was 0.667 (*P* = 0.05), indicating no threshold effect.

MLR demonstrated moderate diagnostic value for DKD. The analysis of MPV revealed a Spearman’s coefficient of -1.000 (*P* = 0.000), confirming a threshold effect; thus, pooled diagnostic estimates were not derived. A meta-analysis was not possible due to the limited number of studies for RDW and SIRI (2 studies each). Details of diagnostic efficacy are listed in **Table 4**.

**Table 4:** Summary of diagnostic value of other inflammatory markers for diabetic kidney disease.

Markers	Number of studies (n)	Sensitivity	Specificity	PLR	NLR	OR	ROC-AUC	Spearman coefficient	*P*
MLR	3	0.63	0.54	2.17	0.45	5.02	0.74	0.500	0.667
RDW	2	0.70	0.65	–	–	–	–	–	–
SIRI	2	0.61	0.59	–	–	–	–	–	–

Substantial heterogeneity persisted for SII and PLR in the diagnosis of DKD. Subgroup analyses were implemented across ten domains to explore sources, including study location (China vs. other), ethnicity (Asian vs. other), control type (T2DM only vs. other), diagnostic criteria (inclusion of UACR, eGFR, single or multiple criteria, biopsy confirmation), study design, and sex distribution. However, none of these were identified as definitive sources of heterogeneity.

### 3.5 Publication bias

**Figure 6:**
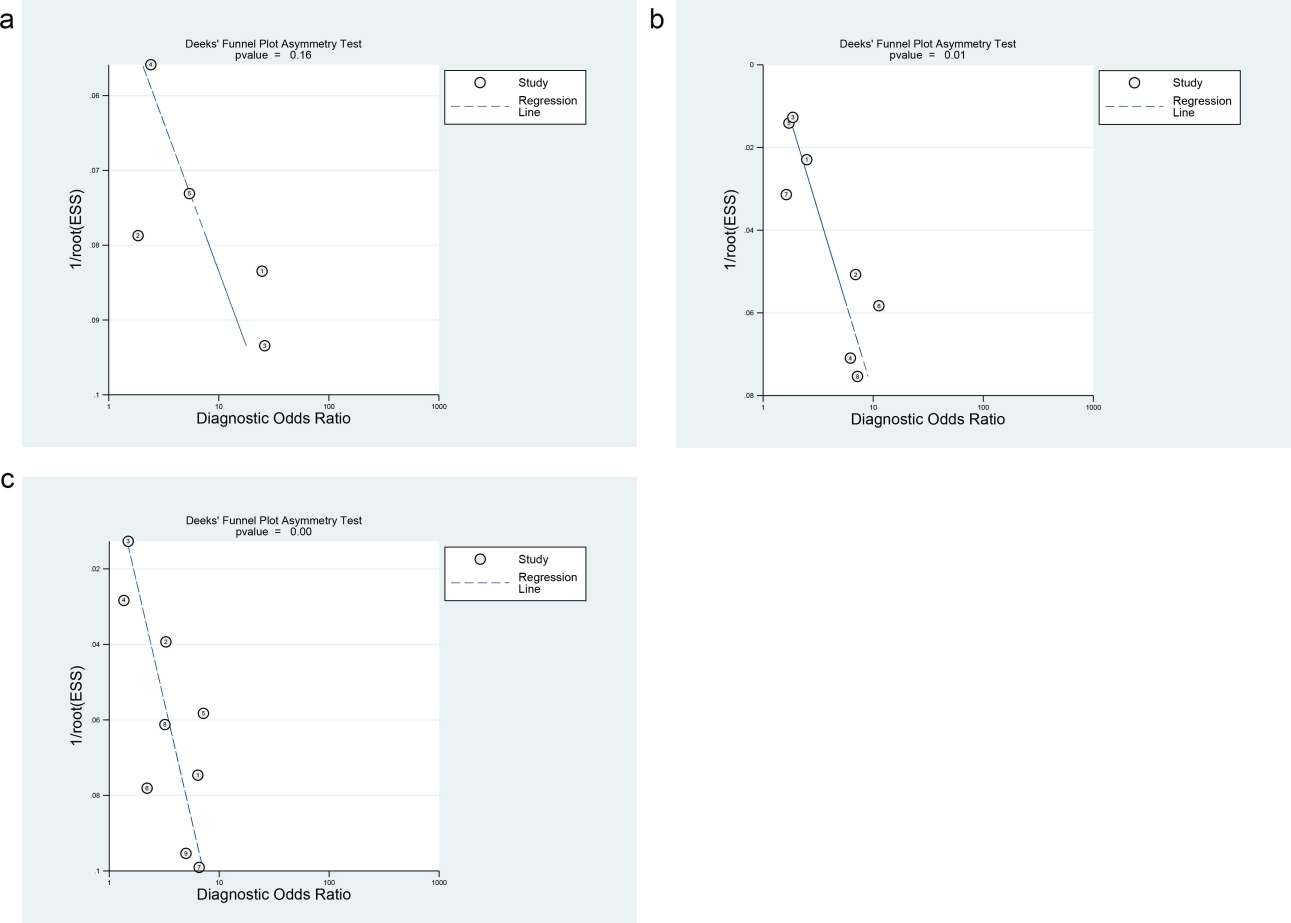
**(a)** Funnel plot of PLR for early DKD diagnosis, **(b)** Funnel plot of SII for DKD diagnosis, **(c)** Funnel plot of PLR for DKD diagnosis.

Funnel plots were generated to evaluate publication bias. No significant bias was detected for PLR in the diagnosis of early DKD (*P* = 0.16). However, potential bias existed for PLR (*P* = 0.00) and SII (*P* = 0.01) in the diagnosis of DKD (**Figure 6**).

## 4 Discussion

DKD is the principal cause of ESRD worldwide, marked by an insidious course and irreversible progression. Early and precise identification is crucial for delaying the decline of renal function, reducing cardiovascular risk, and lowering mortality (53–55). Traditional markers like eGFR and UACR lack sufficient sensitivity for early detection (8, 9), while invasive renal biopsy is costly and carries significant risks, and the routine use of such tests is difficult (7). Consequently, identifying convenient, non-invasive, and efficient early diagnostic biomarkers represents an urgent clinical need.

Mounting evidence positions inflammation as a core driver throughout the progression of DKD, from early activation to advanced fibrosis (10). Routine blood-derived inflammatory markers (PLR, SII, MLR, RDW, etc.) offer advantages of accessibility, low cost, and repeatability, attracting significant research interest. However, their diagnostic performance across different DKD stages, underlying mechanisms, and optimal clinical contexts remains unclear (13–15). Our meta-analysis integrates 32 clinical studies (11 early DKD, 649 subjects; 21 DKD, 9,120 subjects) to systematically evaluate the diagnostic value of six inflammatory markers (SII, PLR, MLR, MPV, RDW, SIRI) and mechanistic links, providing evidence-based guidance for practice.

The meta-analysis confirmed notable variation in diagnostic efficacy among different blood-derived inflammatory markers across DKD stages. For early DKD, PLR showed moderate diagnostic accuracy with an SROC-AUC of 0.78 (95% CI: 0.74-0.81), slightly surpassing MLR and RDW (both AUC = 0.74). PLR also exhibited higher sensitivity (0.74, 95% CI: 0.65-0.81). This suggests its potential as a preferred convenient screening tool for early DKD. MPV displayed a lower diagnostic value (AUC = 0.68) for screening early DKD. In established DKD, SII provided moderate diagnostic accuracy (AUC = 0.71, 95% CI: 0.67-0.74), comparable to MLR (AUC = 0.74), though the diagnostic stability of MLR was influenced by the limited number of eligible studies. The diagnostic performance of PLR was lower in this stage (AUC = 0.68, 95% CI: 0.64-0.72), indicating diminishing value with disease progression. The diagnostic value for MPV in DKD was not analyzed due to the heterogeneity caused by threshold effects. The diagnostic power of MPV needs to be validated by larger multi-center studies. Heterogeneity analysis identified study design as the sole source of heterogeneity in the sensitivity of PLR for diagnosing early DKD. Case-control studies yielded higher sensitivity (0.76, 95% CI: 0.69-0.83) than the single cross-sectional study (0.61, 95% CI: 0.44-0.77), potentially due to stricter sample matching and criteria in case-control designs. Subgroup analyses by ethnicity, diagnostic criteria, and population distribution found no notable influence on heterogeneity for DKD diagnosis. This suggests that the differences in diagnostic performance and heterogeneity likely stem from the distinct pathophysiological mechanisms that each marker reflects.

Renal tissues exhibit a state of sterile chronic inflammation in DKD. Driven by local metabolic disturbances and systemic factors, this inflammation serves as the critical link connecting early metabolic abnormalities, hemodynamic changes, and end-stage renal fibrosis (10). Persistent hyperglycemia activates polyol and hexosamine pathways, facilitates the aggregation of advanced glycation end-products, and induces mitochondrial dysfunction with excessive release of reactive oxygen species (ROS) (56–58). ROS acts as a key inflammatory trigger, activating nuclear factor-κB, the NLRP3 inflammasome, and the cGAS-STING/RIG-I pathway (59). These pathways drive expression of pro-inflammatory and chemotactic factors like IL-1β, TNF-α, and monocyte chemoattractant protein-1 (MCP-1) (56, 57), creating a vicious ‘metabolic dysregulation-immunity activation’ cycle (59). This systemic and local inflammatory burden can be quantified by various routine blood-derived markers. Their abnormalities directly reflect activation of pathological mechanisms. SII integrates neutrophil, platelet, and lymphocyte counts, accurately mirroring ROS-induced systemic inflammatory imbalance (57). Elevated SII indicates enhanced pro-inflammatory activities of neutrophils coupled with suppressed anti-inflammatory function of lymphocytes. These alterations synergistically aggravate local renal immune infiltration (56). This promotes monocyte recruitment and M1 macrophage polarization, accelerating damage to the glomerular basement membrane and disruption of podocyte cytoskeleton (59). Increased PLR signifies enhanced platelet activation under the stimulation of ROS. This process facilitates the release of pro-inflammatory mediators like platelet-derived growth factor (58) and coincides with a weakened lymphocyte anti-inflammatory capacity (59). Together, these processes promote mesangial cell proliferation, extracellular matrix deposition, and tubular epithelial transdifferentiation (56, 58). MLR directly reflects MCP-1-mediated recruitment of monocytes in the kidney and M1 macrophage polarization (57). In high MLR states, polarized macrophages release large quantities of pro-inflammatory factors and ROS, directly injuring glomerular endothelial and tubular epithelial cells (56). They also activate the transforming growth factor-β/Smad pathway, accelerating fibrosis (59). MLR levels are closely correlated with the severity of interstitial fibrosis (57). Elevated RDW arises from impaired erythropoiesis due to chronic DKD-associated inflammation (elevated TNF-α, IL-6) (57). Such impairment increases the heterogeneity of erythrocytes and reduces oxygen-carrying capacity (58). This exacerbates renal tissue hypoxia, which activates the hypoxia-inducible factor-1α pathway. Together, these changes form a secondary ‘inflammation-RWD abnormality-hypoxia-inflammation amplification’ cycle (60). This cycle, in turn, accelerates tubular atrophy and interstitial fibrosis (57, 60). Higher MPV reflects inflammation-mediated (e.g., by TNF-α) platelet activation (56). Activated platelets exhibit enhanced adhesion and aggregation, causing renal microcirculatory impairment and thrombosis (58); they also release mediators that stimulate mesangial cell proliferation (59). These processes collectively aggravate glomerular hypertension and sclerosis. MPV levels are correlated positively with UACR, indirectly reflecting the severity of damage to the glomerular filtration membrane (56, 58). Collectively, these inflammatory markers depict the inflammation-driven pathological landscape of DKD across multiple dimensions: systemic inflammatory imbalance, local immune infiltration, and hematopoietic and platelet metabolic disturbances. Their dysregulation further exacerbates monocyte infiltration, T lymphocyte activation, and complement system dysfunction (56, 57). These dysfunctions cause disruption of podocyte cytoskeleton, endothelial injury, and tubular epithelial transdifferentiation (58, 59). These events ultimately drive the progression to proteinuria, glomerulosclerosis, and interstitial fibrosis (56, 57, 60), thereby constructing a complete pathogenic chain: ‘metabolic disorder-oxidative stress-inflammation initiation-marker abnormality-immune infiltration-tissue injury’ (57–59).

Compared to prior studies on inflammatory markers in DKD, our meta-analysis possesses several strengths. First, it represents the first evidence-based evaluation of the diagnostic value of routine blood-derived inflammatory markers for both early and established DKD. Second, it encompasses more markers, integrating traditional indices (PLR, MLR, MPV, RDW) with novel ones (SII, SIRI) and comparing their diagnostic performance. This addresses the limitations of prior research that primarily focused on individual markers. Finally, subgroup analyses were more refined, strictly differentiating between early DKD and DKD subgroups to clarify stage-specific diagnostic value. Limitations must also be acknowledged. First, a pooled analysis on some markers (RDW, SIRI) was not possible due to insufficient studies, limiting the comprehensiveness of the results. Existing studies predominantly involved Asian populations, lacking data from other ethnicities. Only one study was found in the USA, two in Israel and Syria, six in Turkey, and two in Egypt. No additional studies from Europe or America were found. Therefore, the current meta-analysis does not reflect the applicability of these proposed biomarkers for the early detection of DKD in patients with T2DM in the real world. Second, heterogeneity remained incompletely addressed. The clear sources of high heterogeneity for SII and PLR in DKD were not detected. While threshold effects were excluded, unexamined confounders like assay method differences, population comorbidities, or nuanced diagnostic criteria variations may persist. Third, potential publication bias for PLR and SII in the diagnosis of DKD may cause overestimated performance. Included observational studies also carry inherent risks of selection and information bias. Fourth, mechanistic links are inadequately validated. Our meta-analysis focused on diagnostic performance and did not verify the direct links to core pathological mechanisms of DKD. This gap should be bridged by future mechanistic studies. Given these limitations, the conclusions drawn from this marker remain preliminary and require cautious interpretation in their clinical application.

Future investigations may pursue the following directions. First, the research scale and the diversity of the population should be expanded. More studies focused on SII in early DKD, as well as multi-center, trans-ethnic prospective cohort studies, are required. Second, assays and cut-offs should be standardized. Detection methods and interpretation criteria should be harmonized. Unified diagnostic thresholds should be established to diminish heterogeneity. Third, diagnostic models integrating multiple markers should be constructed. Inflammatory markers should be combined with traditional indices like UACR and eGFR to enhance the diagnostic power for early DKD. Finally, mechanistic and translational research should be conducted. Cellular or animal models should be employed to elucidate molecular mechanisms linking these markers to the progression of DKD so as to strengthen their diagnostic rationale.

## 5 Conclusion

Common immune-inflammatory markers possess diagnostic value for both early and established DKD. PLR represents a convenient screening indicator for early DKD, while SII functions similarly for screening DKD. This analysis also clarifies the diagnostic potential of MLR, RDW, and MPV across DKD stages. This marker system is particularly suitable for early screening in primary care settings and large-scale populations at risk of T2DM, offering a new evidence-based tool for early intervention and disease management. Future multi-center prospective studies should be conducted to verify their clinical utility and guide therapeutic strategies, ultimately aiming to improve prognosis for individuals with T2DM-associated DKD.

## Data Availability

The original contributions presented in the study are included in the article/**Supplementary Material**. Further inquiries can be directed to the corresponding author.

## Supplementary material

Supplementary Table 1 PRISMA+DTA checklist.

Supplementary Table 2 Retrieval strategy.
