# Supplementary material for: The diagnostic value of immune-inflammatory markers for diabetic kidney disease in type 2 diabetic patients: a meta-analysis
Source: Front Endocrinol (Lausanne). 2026 Mar 25;17:1811189. doi: 10.3389/fendo.2026.1811189 (PMC13056621; doi:10.3389/fendo.2026.1811189)
Supplement: Supplementary Table 1 — PRISMA+DTA checklist. [file Table1.doc]

**Table S1 Retrieval strategy**

**Pubmed: 134records**

**SII:**

#1: "Diabetic Nephropathies"[Mesh]

#2: 'diabetes nephropathy'[Title/Abstract] OR 'diabetic kidney disease'[Title/Abstract] OR 'diabetic nephropathies'[Title/Abstract] OR 'diabetic renal disease'[Title/Abstract] OR 'diabetic nephropathy'[Title/Abstract] OR 'Diabetic Kidney Disease'[Title/Abstract] OR 'Diabetic Kidney Diseases'[Title/Abstract] OR 'Diabetic Glomerulosclerosis'[Title/Abstract] OR 'Intracapillary Glomerulosclerosis'[Title/Abstract] OR 'Kimmelstiel Wilson Disease'[Title/Abstract] OR 'Nodular Glomerulosclerosis'[Title/Abstract] OR 'Kimmelstiel Wilson Syndrom'[Title/Abstract]

#3: ("Diabetic Nephropathies"[Mesh]) OR ('diabetes nephropathy'[Title/Abstract] OR 'diabetic kidney disease'[Title/Abstract] OR 'diabetic nephropathies'[Title/Abstract] OR 'diabetic renal disease'[Title/Abstract] OR 'diabetic nephropathy'[Title/Abstract] OR 'Diabetic Kidney Disease'[Title/Abstract] OR 'Diabetic Kidney Diseases'[Title/Abstract] OR 'Diabetic Glomerulosclerosis'[Title/Abstract] OR 'Intracapillary Glomerulosclerosis'[Title/Abstract] OR 'Kimmelstiel Wilson Disease'[Title/Abstract] OR 'Nodular Glomerulosclerosis'[Title/Abstract] OR 'Kimmelstiel Wilson Syndrom'[Title/Abstract])

#4: 'systemic immune-inflammation index'[Title/Abstract] OR 'systemic immune-inflammatory index'[Title/Abstract] OR 'Systemic Immunity-inflammation Index'[Title/Abstract] OR 'systemic immune inflammation index'[Title/Abstract] OR 'systemic immune inflammatory index'[Title/Abstract] OR 'SII'[Title/Abstract] OR 'neutrophil × platelets/ lymphocyte'[Title/Abstract]

#5: ((Diabetic Nephropathies[MeSH Terms]) OR ('diabetes nephropathy'[Title/Abstract] OR 'diabetic kidney disease'[Title/Abstract] OR 'diabetic nephropathies'[Title/Abstract] OR 'diabetic renal disease'[Title/Abstract] OR 'diabetic nephropathy'[Title/Abstract] OR 'Diabetic Kidney Disease'[Title/Abstract] OR 'Diabetic Kidney Diseases'[Title/Abstract] OR 'Diabetic Glomerulosclerosis'[Title/Abstract] OR 'Intracapillary Glomerulosclerosis'[Title/Abstract] OR 'Kimmelstiel Wilson Disease'[Title/Abstract] OR 'Nodular Glomerulosclerosis'[Title/Abstract] OR 'Kimmelstiel Wilson Syndrom'[Title/Abstract])) AND ('systemic immune-inflammation index'[Title/Abstract] OR 'systemic immune-inflammatory index'[Title/Abstract] OR 'Systemic Immunity-inflammation Index'[Title/Abstract] OR 'systemic immune inflammation index'[Title/Abstract] OR 'systemic immune inflammatory index'[Title/Abstract] OR 'SII'[Title/Abstract] OR 'neutrophil × platelets/ lymphocyte'[Title/Abstract])

**PLR:**

#1: "Diabetic Nephropathies"[Mesh]

#2: 'diabetes nephropathy'[Title/Abstract] OR 'diabetic kidney disease'[Title/Abstract] OR 'diabetic nephropathies'[Title/Abstract] OR 'diabetic renal disease'[Title/Abstract] OR 'diabetic nephropathy'[Title/Abstract] OR 'Diabetic Kidney Disease'[Title/Abstract] OR 'Diabetic Kidney Diseases'[Title/Abstract] OR 'Diabetic Glomerulosclerosis'[Title/Abstract] OR 'Intracapillary Glomerulosclerosis'[Title/Abstract] OR 'Kimmelstiel Wilson Disease'[Title/Abstract] OR 'Nodular Glomerulosclerosis'[Title/Abstract] OR 'Kimmelstiel Wilson Syndrom'[Title/Abstract]

#3: ("Diabetic Nephropathies"[Mesh]) OR ('diabetes nephropathy'[Title/Abstract] OR 'diabetic kidney disease'[Title/Abstract] OR 'diabetic nephropathies'[Title/Abstract] OR 'diabetic renal disease'[Title/Abstract] OR 'diabetic nephropathy'[Title/Abstract] OR 'Diabetic Kidney Disease'[Title/Abstract] OR 'Diabetic Kidney Diseases'[Title/Abstract] OR 'Diabetic Glomerulosclerosis'[Title/Abstract] OR 'Intracapillary Glomerulosclerosis'[Title/Abstract] OR 'Kimmelstiel Wilson Disease'[Title/Abstract] OR 'Nodular Glomerulosclerosis'[Title/Abstract] OR 'Kimmelstiel Wilson Syndrom'[Title/Abstract])

#4: 'platelet to lymphocyte ratio'[Title/Abstract] OR 'platelet/lymphocyte ratio'[Title/Abstract] OR 'PLR'[Title/Abstract] OR 'thrombocyte lymphocyte ratio'[Title/Abstract] OR 'platelet lymphocyte ratio'[Title/Abstract] OR 'platelet/lymphocyte'[Title/Abstract]

#5: ((Diabetic Nephropathies[MeSH Terms]) OR ('diabetes nephropathy'[Title/Abstract] OR 'diabetic kidney disease'[Title/Abstract] OR 'diabetic nephropathies'[Title/Abstract] OR 'diabetic renal disease'[Title/Abstract] OR 'diabetic nephropathy'[Title/Abstract] OR 'Diabetic Kidney Disease'[Title/Abstract] OR 'Diabetic Kidney Diseases'[Title/Abstract] OR 'Diabetic Glomerulosclerosis'[Title/Abstract] OR 'Intracapillary Glomerulosclerosis'[Title/Abstract] OR 'Kimmelstiel Wilson Disease'[Title/Abstract] OR 'Nodular Glomerulosclerosis'[Title/Abstract] OR 'Kimmelstiel Wilson Syndrom'[Title/Abstract])) AND ('platelet to lymphocyte ratio'[Title/Abstract] OR 'platelet/lymphocyte ratio'[Title/Abstract] OR 'PLR'[Title/Abstract] OR 'thrombocyte lymphocyte ratio'[Title/Abstract] OR 'platelet lymphocyte ratio'[Title/Abstract] OR 'platelet/lymphocyte'[Title/Abstract])

**MLR:**

#1: "Diabetic Nephropathies"[Mesh]

#2: 'diabetes nephropathy'[Title/Abstract] OR 'diabetic kidney disease'[Title/Abstract] OR 'diabetic nephropathies'[Title/Abstract] OR 'diabetic renal disease'[Title/Abstract] OR 'diabetic nephropathy'[Title/Abstract] OR 'Diabetic Kidney Disease'[Title/Abstract] OR 'Diabetic Kidney Diseases'[Title/Abstract] OR 'Diabetic Glomerulosclerosis'[Title/Abstract] OR 'Intracapillary Glomerulosclerosis'[Title/Abstract] OR 'Kimmelstiel Wilson Disease'[Title/Abstract] OR 'Nodular Glomerulosclerosis'[Title/Abstract] OR 'Kimmelstiel Wilson Syndrom'[Title/Abstract]

#3: ("Diabetic Nephropathies"[Mesh]) OR ('diabetes nephropathy'[Title/Abstract] OR 'diabetic kidney disease'[Title/Abstract] OR 'diabetic nephropathies'[Title/Abstract] OR 'diabetic renal disease'[Title/Abstract] OR 'diabetic nephropathy'[Title/Abstract] OR 'Diabetic Kidney Disease'[Title/Abstract] OR 'Diabetic Kidney Diseases'[Title/Abstract] OR 'Diabetic Glomerulosclerosis'[Title/Abstract] OR 'Intracapillary Glomerulosclerosis'[Title/Abstract] OR 'Kimmelstiel Wilson Disease'[Title/Abstract] OR 'Nodular Glomerulosclerosis'[Title/Abstract] OR 'Kimmelstiel Wilson Syndrom'[Title/Abstract])

#4: monocyte to lymphocyte ratio'[Title/Abstract] OR 'monocyte/lymphocyte ratio'[Title/Abstract] OR 'monocyte lymphocyte ratio'[Title/Abstract] OR 'lymphocyte to monocyte ratio'[Title/Abstract] OR 'lymphocyte monocyte ratio'[Title/Abstract] OR 'lymphocyte/monocyte ratio'[Title/Abstract] OR 'MLR'[Title/Abstract] OR 'LMR'[Title/Abstract]

#5: ((Diabetic Nephropathies[MeSH Terms]) OR ('diabetes nephropathy'[Title/Abstract] OR 'diabetic kidney disease'[Title/Abstract] OR 'diabetic nephropathies'[Title/Abstract] OR 'diabetic renal disease'[Title/Abstract] OR 'diabetic nephropathy'[Title/Abstract] OR 'Diabetic Kidney Disease'[Title/Abstract] OR 'Diabetic Kidney Diseases'[Title/Abstract] OR 'Diabetic Glomerulosclerosis'[Title/Abstract] OR 'Intracapillary Glomerulosclerosis'[Title/Abstract] OR 'Kimmelstiel Wilson Disease'[Title/Abstract] OR 'Nodular Glomerulosclerosis'[Title/Abstract] OR 'Kimmelstiel Wilson Syndrom'[Title/Abstract])) AND ('monocyte to lymphocyte ratio'[Title/Abstract] OR 'monocyte/lymphocyte ratio'[Title/Abstract] OR 'monocyte lymphocyte ratio'[Title/Abstract] OR 'lymphocyte to monocyte ratio'[Title/Abstract] OR 'lymphocyte monocyte ratio'[Title/Abstract] OR 'lymphocyte/monocyte ratio'[Title/Abstract] OR 'MLR'[Title/Abstract] OR 'LMR'[Title/Abstract])

**MPV:**

#1: "Diabetic Nephropathies"[Mesh]

#2: 'diabetes nephropathy'[Title/Abstract] OR 'diabetic kidney disease'[Title/Abstract] OR 'diabetic nephropathies'[Title/Abstract] OR 'diabetic renal disease'[Title/Abstract] OR 'diabetic nephropathy'[Title/Abstract] OR 'Diabetic Kidney Disease'[Title/Abstract] OR 'Diabetic Kidney Diseases'[Title/Abstract] OR 'Diabetic Glomerulosclerosis'[Title/Abstract] OR 'Intracapillary Glomerulosclerosis'[Title/Abstract] OR 'Kimmelstiel Wilson Disease'[Title/Abstract] OR 'Nodular Glomerulosclerosis'[Title/Abstract] OR 'Kimmelstiel Wilson Syndrom'[Title/Abstract]

#3: ("Diabetic Nephropathies"[Mesh]) OR ('diabetes nephropathy'[Title/Abstract] OR 'diabetic kidney disease'[Title/Abstract] OR 'diabetic nephropathies'[Title/Abstract] OR 'diabetic renal disease'[Title/Abstract] OR 'diabetic nephropathy'[Title/Abstract] OR 'Diabetic Kidney Disease'[Title/Abstract] OR 'Diabetic Kidney Diseases'[Title/Abstract] OR 'Diabetic Glomerulosclerosis'[Title/Abstract] OR 'Intracapillary Glomerulosclerosis'[Title/Abstract] OR 'Kimmelstiel Wilson Disease'[Title/Abstract] OR 'Nodular Glomerulosclerosis'[Title/Abstract] OR 'Kimmelstiel Wilson Syndrom'[Title/Abstract])

#4: Mean Platelet Volume[MeSH Terms]

#5: 'average platelet volume'[Title/Abstract] OR 'average thrombocyte volume'[Title/Abstract] OR 'mean thrombocyte volume'[Title/Abstract] OR 'mean platelet volume'[Title/Abstract] OR 'Mean Platelet Volumes'[Title/Abstract] OR 'MPV'[Title/Abstract]

#6: (Mean Platelet Volume[MeSH Terms]) OR ('average platelet volume'[Title/Abstract] OR 'average thrombocyte volume'[Title/Abstract] OR 'mean thrombocyte volume'[Title/Abstract] OR 'mean platelet volume'[Title/Abstract] OR 'Mean Platelet Volumes'[Title/Abstract] OR 'MPV'[Title/Abstract])

#7: ((Diabetic Nephropathies[MeSH Terms]) OR ('diabetes nephropathy'[Title/Abstract] OR 'diabetic kidney disease'[Title/Abstract] OR 'diabetic nephropathies'[Title/Abstract] OR 'diabetic renal disease'[Title/Abstract] OR 'diabetic nephropathy'[Title/Abstract] OR 'Diabetic Kidney Disease'[Title/Abstract] OR 'Diabetic Kidney Diseases'[Title/Abstract] OR 'Diabetic Glomerulosclerosis'[Title/Abstract] OR 'Intracapillary Glomerulosclerosis'[Title/Abstract] OR 'Kimmelstiel Wilson Disease'[Title/Abstract] OR 'Nodular Glomerulosclerosis'[Title/Abstract] OR 'Kimmelstiel Wilson Syndrom'[Title/Abstract])) AND ((Mean Platelet Volume[MeSH Terms]) OR ('average platelet volume'[Title/Abstract] OR 'average thrombocyte volume'[Title/Abstract] OR 'mean thrombocyte volume'[Title/Abstract] OR 'mean platelet volume'[Title/Abstract] OR 'Mean Platelet Volumes'[Title/Abstract] OR 'MPV'[Title/Abstract]))

**RDW:**

#1: "Diabetic Nephropathies"[Mesh]

#2: 'diabetes nephropathy'[Title/Abstract] OR 'diabetic kidney disease'[Title/Abstract] OR 'diabetic nephropathies'[Title/Abstract] OR 'diabetic renal disease'[Title/Abstract] OR 'diabetic nephropathy'[Title/Abstract] OR 'Diabetic Kidney Disease'[Title/Abstract] OR 'Diabetic Kidney Diseases'[Title/Abstract] OR 'Diabetic Glomerulosclerosis'[Title/Abstract] OR 'Intracapillary Glomerulosclerosis'[Title/Abstract] OR 'Kimmelstiel Wilson Disease'[Title/Abstract] OR 'Nodular Glomerulosclerosis'[Title/Abstract] OR 'Kimmelstiel Wilson Syndrom'[Title/Abstract]

#3: ("Diabetic Nephropathies"[Mesh]) OR ('diabetes nephropathy'[Title/Abstract] OR 'diabetic kidney disease'[Title/Abstract] OR 'diabetic nephropathies'[Title/Abstract] OR 'diabetic renal disease'[Title/Abstract] OR 'diabetic nephropathy'[Title/Abstract] OR 'Diabetic Kidney Disease'[Title/Abstract] OR 'Diabetic Kidney Diseases'[Title/Abstract] OR 'Diabetic Glomerulosclerosis'[Title/Abstract] OR 'Intracapillary Glomerulosclerosis'[Title/Abstract] OR 'Kimmelstiel Wilson Disease'[Title/Abstract] OR 'Nodular Glomerulosclerosis'[Title/Abstract] OR 'Kimmelstiel Wilson Syndrom'[Title/Abstract])

#4: "Erythrocyte Indices"[Mesh]

#5: 'erythrocyte distribution width'[Title/Abstract] OR 'RCDW'[Title/Abstract] OR 'RDW'[Title/Abstract] OR 'RDW-CV'[Title/Abstract] OR 'RDW-SD'[Title/Abstract] OR 'red blood cell distribution width'[Title/Abstract] OR 'Red Cell Indexes'[Title/Abstract] OR 'Erythrocyte Index'[Title/Abstract] OR 'Erythrocyte Indexes'[Title/Abstract] OR 'Red Cell Index'[Title/Abstract] OR 'Red Cell Indices'[Title/Abstract] OR 'Erythrocyte Size Determination'[Title/Abstract] OR 'Erythrocyte Size Determinations'[Title/Abstract] OR 'Mean Corpuscular Volume'[Title/Abstract] OR 'Mean Corpuscular Volumes'[Title/Abstract] OR 'Mean Cell Volume'[Title/Abstract] OR 'Mean Cell Volumes'[Title/Abstract] OR 'Mean Corpuscular Hemoglobin'[Title/Abstract] OR 'Mean Corpuscular Hemoglobins'[Title/Abstract] OR 'Erythrocyte Thickness'[Title/Abstract] OR 'Erythrocyte Diameter'[Title/Abstract] OR 'Erythrocyte Diameters'[Title/Abstract] OR 'Mean Corpuscular Hemoglobulin Concentration'[Title/Abstract] OR 'Mean Cell Hemoglobin Concentration'[Title/Abstract] OR 'Red Cell Distribution Width'[Title/Abstract] OR 'erythrocyte indices'[Title/Abstract]

#6: ("Erythrocyte Indices"[Mesh]) OR ('erythrocyte distribution width'[Title/Abstract] OR 'RCDW'[Title/Abstract] OR 'RDW'[Title/Abstract] OR 'RDW-CV'[Title/Abstract] OR 'RDW-SD'[Title/Abstract] OR 'red blood cell distribution width'[Title/Abstract] OR 'Red Cell Indexes'[Title/Abstract] OR 'Erythrocyte Index'[Title/Abstract] OR 'Erythrocyte Indexes'[Title/Abstract] OR 'Red Cell Index'[Title/Abstract] OR 'Red Cell Indices'[Title/Abstract] OR 'Erythrocyte Size Determination'[Title/Abstract] OR 'Erythrocyte Size Determinations'[Title/Abstract] OR 'Mean Corpuscular Volume'[Title/Abstract] OR 'Mean Corpuscular Volumes'[Title/Abstract] OR 'Mean Cell Volume'[Title/Abstract] OR 'Mean Cell Volumes'[Title/Abstract] OR 'Mean Corpuscular Hemoglobin'[Title/Abstract] OR 'Mean Corpuscular Hemoglobins'[Title/Abstract] OR 'Erythrocyte Thickness'[Title/Abstract] OR 'Erythrocyte Diameter'[Title/Abstract] OR 'Erythrocyte Diameters'[Title/Abstract] OR 'Mean Corpuscular Hemoglobulin Concentration'[Title/Abstract] OR 'Mean Cell Hemoglobin Concentration'[Title/Abstract] OR 'Red Cell Distribution Width'[Title/Abstract] OR 'erythrocyte indices'[Title/Abstract])

#7: (("Diabetic Nephropathies"[Mesh]) OR ('diabetes nephropathy'[Title/Abstract] OR 'diabetic kidney disease'[Title/Abstract] OR 'diabetic nephropathies'[Title/Abstract] OR 'diabetic renal disease'[Title/Abstract] OR 'diabetic nephropathy'[Title/Abstract] OR 'Diabetic Kidney Disease'[Title/Abstract] OR 'Diabetic Kidney Diseases'[Title/Abstract] OR 'Diabetic Glomerulosclerosis'[Title/Abstract] OR 'Intracapillary Glomerulosclerosis'[Title/Abstract] OR 'Kimmelstiel Wilson Disease'[Title/Abstract] OR 'Nodular Glomerulosclerosis'[Title/Abstract] OR 'Kimmelstiel Wilson Syndrom'[Title/Abstract])) AND (("Erythrocyte Indices"[Mesh]) OR ('erythrocyte distribution width'[Title/Abstract] OR 'RCDW'[Title/Abstract] OR 'RDW'[Title/Abstract] OR 'RDW-CV'[Title/Abstract] OR 'RDW-SD'[Title/Abstract] OR 'red blood cell distribution width'[Title/Abstract] OR 'Red Cell Indexes'[Title/Abstract] OR 'Erythrocyte Index'[Title/Abstract] OR 'Erythrocyte Indexes'[Title/Abstract] OR 'Red Cell Index'[Title/Abstract] OR 'Red Cell Indices'[Title/Abstract] OR 'Erythrocyte Size Determination'[Title/Abstract] OR 'Erythrocyte Size Determinations'[Title/Abstract] OR 'Mean Corpuscular Volume'[Title/Abstract] OR 'Mean Corpuscular Volumes'[Title/Abstract] OR 'Mean Cell Volume'[Title/Abstract] OR 'Mean Cell Volumes'[Title/Abstract] OR 'Mean Corpuscular Hemoglobin'[Title/Abstract] OR 'Mean Corpuscular Hemoglobins'[Title/Abstract] OR 'Erythrocyte Thickness'[Title/Abstract] OR 'Erythrocyte Diameter'[Title/Abstract] OR 'Erythrocyte Diameters'[Title/Abstract] OR 'Mean Corpuscular Hemoglobulin Concentration'[Title/Abstract] OR 'Mean Cell Hemoglobin Concentration'[Title/Abstract] OR 'Red Cell Distribution Width'[Title/Abstract] OR 'erythrocyte indices'[Title/Abstract]))

**SIRI:**

#1: "Diabetic Nephropathies"[Mesh]

#2: 'diabetes nephropathy'[Title/Abstract] OR 'diabetic kidney disease'[Title/Abstract] OR 'diabetic nephropathies'[Title/Abstract] OR 'diabetic renal disease'[Title/Abstract] OR 'diabetic nephropathy'[Title/Abstract] OR 'Diabetic Kidney Disease'[Title/Abstract] OR 'Diabetic Kidney Diseases'[Title/Abstract] OR 'Diabetic Glomerulosclerosis'[Title/Abstract] OR 'Intracapillary Glomerulosclerosis'[Title/Abstract] OR 'Kimmelstiel Wilson Disease'[Title/Abstract] OR 'Nodular Glomerulosclerosis'[Title/Abstract] OR 'Kimmelstiel Wilson Syndrom'[Title/Abstract]

#3: ("Diabetic Nephropathies"[Mesh]) OR ('diabetes nephropathy'[Title/Abstract] OR 'diabetic kidney disease'[Title/Abstract] OR 'diabetic nephropathies'[Title/Abstract] OR 'diabetic renal disease'[Title/Abstract] OR 'diabetic nephropathy'[Title/Abstract] OR 'Diabetic Kidney Disease'[Title/Abstract] OR 'Diabetic Kidney Diseases'[Title/Abstract] OR 'Diabetic Glomerulosclerosis'[Title/Abstract] OR 'Intracapillary Glomerulosclerosis'[Title/Abstract] OR 'Kimmelstiel Wilson Disease'[Title/Abstract] OR 'Nodular Glomerulosclerosis'[Title/Abstract] OR 'Kimmelstiel Wilson Syndrom'[Title/Abstract])

#4: 'systemic inflammation reaction index'[Title/Abstract] OR 'systemic inflammation response index'[Title/Abstract] OR 'systemic inflammatory reaction index'[Title/Abstract] OR 'Systemic Inflammatory Response Index'[Title/Abstract] OR 'SIRI'[Title/Abstract]V

#5: ((Diabetic Nephropathies[MeSH Terms]) OR ('diabetes nephropathy'[Title/Abstract] OR 'diabetic kidney disease'[Title/Abstract] OR 'diabetic nephropathies'[Title/Abstract] OR 'diabetic renal disease'[Title/Abstract] OR 'diabetic nephropathy'[Title/Abstract] OR 'Diabetic Kidney Disease'[Title/Abstract] OR 'Diabetic Kidney Diseases'[Title/Abstract] OR 'Diabetic Glomerulosclerosis'[Title/Abstract] OR 'Intracapillary Glomerulosclerosis'[Title/Abstract] OR 'Kimmelstiel Wilson Disease'[Title/Abstract] OR 'Nodular Glomerulosclerosis'[Title/Abstract] OR 'Kimmelstiel Wilson Syndrom'[Title/Abstract])) AND ('systemic inflammation reaction index'[Title/Abstract] OR 'systemic inflammation response index'[Title/Abstract] OR 'systemic inflammatory reaction index'[Title/Abstract] OR 'Systemic Inflammatory Response Index'[Title/Abstract] OR 'SIRI'[Title/Abstract])

**Embase: 310records**

**SII:**

#1：'diabetic nephropathy'/exp

#2：'diabetic kidney diseases':ab,ti OR 'diabetic nephropathy':ab,ti OR 'diabetic glomerulosclerosis':ab,ti OR 'intracapillary glomerulosclerosis':ab,ti OR 'kimmelstiel wilson disease':ab,ti OR 'nodular glomerulosclerosis':ab,ti OR 'kimmelstiel wilson syndrome':ab,ti OR 'diabetes nephropathy':ab,ti OR 'diabetic kidney disease':ab,ti OR 'diabetic nephropathies':ab,ti OR 'diabetic renal disease':ab,ti

#3: #1 OR #2

#4: 'systemic immune inflammation index'/exp

#5: 'systemic immune-inflammation index':ab,ti OR 'systemic immune-inflammatory index':ab,ti OR 'systemic immunity-inflammation index':ab,ti OR 'systemic immune inflammation index':ab,ti OR 'systemic immune inflammatory index':ab,ti OR 'siis':ab,ti OR 'neutrophil × platelets/ lymphocyte':ab,ti

#6：#4 OR #5

#7：#3 AND #6

**PLR:**

#1：'diabetic nephropathy'/exp

#2: 'diabetic kidney diseases':ab,ti OR 'diabetic nephropathy':ab,ti OR 'diabetic glomerulosclerosis':ab,ti OR 'intracapillary glomerulosclerosis':ab,ti OR 'kimmelstiel wilson disease':ab,ti OR 'nodular glomerulosclerosis':ab,ti OR 'kimmelstiel wilson syndrome':ab,ti OR 'diabetes nephropathy':ab,ti OR 'diabetic kidney disease':ab,ti OR 'diabetic nephropathies':ab,ti OR 'diabetic renal disease':ab,ti

#3: #1 OR #2

#4: 'platelet lymphocyte ratio'/exp

#5: 'platelet to lymphocyte ratio':ab,ti OR 'platelet/lymphocyte ratio':ab,ti OR 'plr':ab,ti OR 'thrombocyte lymphocyte ratio':ab,ti OR 'platelet lymphocyte ratio':ab,ti OR 'platelet/lymphocyte':ab,ti

#6: #4 OR #5

#7: #3 AND #6

**MLR:**

#1：'diabetic nephropathy'/exp

#2: 'diabetic kidney diseases':ab,ti OR 'diabetic nephropathy':ab,ti OR 'diabetic glomerulosclerosis':ab,ti OR 'intracapillary glomerulosclerosis':ab,ti OR 'kimmelstiel wilson disease':ab,ti OR 'nodular glomerulosclerosis':ab,ti OR 'kimmelstiel wilson syndrome':ab,ti OR 'diabetes nephropathy':ab,ti OR 'diabetic kidney disease':ab,ti OR 'diabetic nephropathies':ab,ti OR 'diabetic renal disease':ab,ti

#3: #1 OR #2

#4: 'monocyte lymphocyte ratio'/exp

#5: 'monocyte to lymphocyte ratio':ab,ti OR 'monocyte/lymphocyte ratio':ab,ti OR 'monocyte lymphocyte ratio':ab,ti OR 'lymphocyte to?monocyte?ratio':ab,ti OR 'lymphocyte monocyte?ratio':ab,ti OR 'lymphocyte/monocyte?ratio':ab,ti OR 'lmr':ab,ti OR 'mlr':ab,ti

#6: #4 OR #5

#7: #3 AND #6

**MPV:**

#1：'diabetic nephropathy'/exp

#2: 'diabetic kidney diseases':ab,ti OR 'diabetic nephropathy':ab,ti OR 'diabetic glomerulosclerosis':ab,ti OR 'intracapillary glomerulosclerosis':ab,ti OR 'kimmelstiel wilson disease':ab,ti OR 'nodular glomerulosclerosis':ab,ti OR 'kimmelstiel wilson syndrome':ab,ti OR 'diabetes nephropathy':ab,ti OR 'diabetic kidney disease':ab,ti OR 'diabetic nephropathies':ab,ti OR 'diabetic renal disease':ab,ti

#3: #1 OR #2

#4: 'mean platelet volume'/exp

#5: 'average platelet volume':ab,ti OR 'average thrombocyte volume':ab,ti OR 'mean thrombocyte volume':ab,ti OR 'mean platelet volume':ab,ti OR 'mean platelet volumes':ab,ti OR 'mpv':ab,ti

#6: #4 OR #5

#7: #3 AND #6

**RDW:**

#1：'diabetic nephropathy'/exp

#2: 'diabetic kidney diseases':ab,ti OR 'diabetic nephropathy':ab,ti OR 'diabetic glomerulosclerosis':ab,ti OR 'intracapillary glomerulosclerosis':ab,ti OR 'kimmelstiel wilson disease':ab,ti OR 'nodular glomerulosclerosis':ab,ti OR 'kimmelstiel wilson syndrome':ab,ti OR 'diabetes nephropathy':ab,ti OR 'diabetic kidney disease':ab,ti OR 'diabetic nephropathies':ab,ti OR 'diabetic renal disease':ab,ti

#3: #1 OR #2

#4: 'red blood cell distribution width'/exp

#5: 'erythrocyte distribution width':ab,ti OR 'rcdw':ab,ti OR 'rdw':ab,ti OR 'rdw-cv':ab,ti OR 'rdw-sd':ab,ti OR 'red blood cell distribution width':ab,ti OR 'red cell indexes':ab,ti OR 'erythrocyte index':ab,ti OR 'erythrocyte indexes':ab,ti OR 'red cell index':ab,ti OR 'red cell indices':ab,ti OR 'erythrocyte size determination':ab,ti OR 'erythrocyte size determinations':ab,ti OR 'mean corpuscular volume':ab,ti OR 'mean corpuscular volumes':ab,ti OR 'mean cell volume':ab,ti OR 'mean cell volumes':ab,ti OR 'mean corpuscular hemoglobin':ab,ti OR 'mean corpuscular hemoglobins':ab,ti OR 'erythrocyte thickness':ab,ti OR 'erythrocyte diameter':ab,ti OR 'erythrocyte diameters':ab,ti OR 'mean corpuscular hemoglobulin concentration':ab,ti OR 'mean cell hemoglobin concentration':ab,ti OR 'red cell distribution width':ab,ti OR 'erythrocyte indices':ab,ti

#6: #4 OR #5

#7: #3 AND #6

**SIRI:**

#1：diabetic nephropathy'/exp

#2: 'diabetic kidney diseases':ab,ti OR 'diabetic nephropathy':ab,ti OR 'diabetic glomerulosclerosis':ab,ti OR 'intracapillary glomerulosclerosis':ab,ti OR 'kimmelstiel wilson disease':ab,ti OR 'nodular glomerulosclerosis':ab,ti OR 'kimmelstiel wilson syndrome':ab,ti OR 'diabetes nephropathy':ab,ti OR 'diabetic kidney disease':ab,ti OR 'diabetic nephropathies':ab,ti OR 'diabetic renal disease':ab,ti

#3: #1 OR #2

#4: 'systemic inflammation response index'/exp

#5: 'systemic inflammation reaction index' OR 'systemic inflammation response index (siri)' OR 'systemic inflammatory reaction index' OR 'systemic inflammatory response index' OR 'systemic inflammation response index'

#6: #4 OR #5

#7: #3 AND #6

**Cochrane Library: 46 records**

**SII:**

#1：MeSH descriptor: [Diabetic Nephropathies] explode all trees

#2：(Diabetic Kidney Disease):ti,ab,kw OR (Diabetic Kidney Diseases):ti,ab,kw OR (Diabetic Nephropathy):ti,ab,kw OR (Diabetic Glomerulosclerosis):ti,ab,kw OR (Intracapillary Glomerulosclerosis):ti,ab,kw OR (Kimmelstiel Wilson Disease):ti,ab,kw OR (Nodular Glomerulosclerosis):ti,ab,kw OR (Kimmelstiel Wilson Syndrome):ti,ab,kw OR (diabetes nephropathy):ti,ab,kw OR (diabetic kidney disease):ti,ab,kw OR (diabetic nephropathies):ti,ab,kw OR (diabetic renal disease):ti,ab,kw

#3：#1 OR #2

#4: (systemic immune-inflammation index):ti,ab,kw OR (systemic immune-inflammatory index):ti,ab,kw OR (Systemic Immunity-inflammation Index):ti,ab,kw OR (systemic immune inflammation index):ti,ab,kw OR (systemic immune inflammatory index):ti,ab,kw OR (SII):ti,ab,kw OR (neutrophil × platelets lymphocyte):ti,ab,kw

#5: #3 AND #4

**PLR:**

#1：MeSH descriptor: [Diabetic Nephropathies] explode all trees

#2：(Diabetic Kidney Disease):ti,ab,kw OR (Diabetic Kidney Diseases):ti,ab,kw OR (Diabetic Nephropathy):ti,ab,kw OR (Diabetic Glomerulosclerosis):ti,ab,kw OR (Intracapillary Glomerulosclerosis):ti,ab,kw OR (Kimmelstiel Wilson Disease):ti,ab,kw OR (Nodular Glomerulosclerosis):ti,ab,kw OR (Kimmelstiel Wilson Syndrome):ti,ab,kw OR (diabetes nephropathy):ti,ab,kw OR (diabetic kidney disease):ti,ab,kw OR (diabetic nephropathies):ti,ab,kw OR (diabetic renal disease):ti,ab,kw

#3：#1 OR #2

#4: (platelet to lymphocyte ratio):ti,ab,kw OR (PLR):ti,ab,kw OR (thrombocyte lymphocyte ratio):ti,ab,kw OR (platelet lymphocyte ratio):ti,ab,kw OR (platelet lymphocyte):ti,ab,kw

#5: #3 AND #4

**MLR:**

#1：MeSH descriptor: [Diabetic Nephropathies] explode all trees

#2：(Diabetic Kidney Disease):ti,ab,kw OR (Diabetic Kidney Diseases):ti,ab,kw OR (Diabetic Nephropathy):ti,ab,kw OR (Diabetic Glomerulosclerosis):ti,ab,kw OR (Intracapillary Glomerulosclerosis):ti,ab,kw OR (Kimmelstiel Wilson Disease):ti,ab,kw OR (Nodular Glomerulosclerosis):ti,ab,kw OR (Kimmelstiel Wilson Syndrome):ti,ab,kw OR (diabetes nephropathy):ti,ab,kw OR (diabetic kidney disease):ti,ab,kw OR (diabetic nephropathies):ti,ab,kw OR (diabetic renal disease):ti,ab,kw

#3：#1 OR #2

#4: (monocyte to lymphocyte ratio):ti,ab,kw OR (monocyte lymphocyte ratio):ti,ab,kw OR (lymphocyte to monocyte ratio):ti,ab,kw OR (lymphocyte monocyte ratio):ti,ab,kw OR (LMR):ti,ab,kw OR (MLR):ti,ab,kw

#5: #3 AND #4

**MPV:**

#1：MeSH descriptor: [Diabetic Nephropathies] explode all trees

#2：(Diabetic Kidney Disease):ti,ab,kw OR (Diabetic Kidney Diseases):ti,ab,kw OR (Diabetic Nephropathy):ti,ab,kw OR (Diabetic Glomerulosclerosis):ti,ab,kw OR (Intracapillary Glomerulosclerosis):ti,ab,kw OR (Kimmelstiel Wilson Disease):ti,ab,kw OR (Nodular Glomerulosclerosis):ti,ab,kw OR (Kimmelstiel Wilson Syndrome):ti,ab,kw OR (diabetes nephropathy):ti,ab,kw OR (diabetic kidney disease):ti,ab,kw OR (diabetic nephropathies):ti,ab,kw OR (diabetic renal disease):ti,ab,kw

#3: #1 OR #2

#4: MeSH descriptor: [Mean Platelet Volume] explode all trees

#5: (average platelet volume):ti,ab,kw OR (average thrombocyte volume):ti,ab,kw OR (mean thrombocyte volume):ti,ab,kw OR (mean platelet volume):ti,ab,kw OR (Mean Platelet Volumes):ti,ab,kw OR (MPV):ti,ab,kw

#6: #4 OR #5

#7: #3 AND #6

**RDW:**

#1：MeSH descriptor: [Diabetic Nephropathies] explode all trees

#2：(Diabetic Kidney Disease):ti,ab,kw OR (Diabetic Kidney Diseases):ti,ab,kw OR (Diabetic Nephropathy):ti,ab,kw OR (Diabetic Glomerulosclerosis):ti,ab,kw OR (Intracapillary Glomerulosclerosis):ti,ab,kw OR (Kimmelstiel Wilson Disease):ti,ab,kw OR (Nodular Glomerulosclerosis):ti,ab,kw OR (Kimmelstiel Wilson Syndrome):ti,ab,kw OR (diabetes nephropathy):ti,ab,kw OR (diabetic kidney disease):ti,ab,kw OR (diabetic nephropathies):ti,ab,kw OR (diabetic renal disease):ti,ab,kw

#3: #1 OR #2

#4: MeSH descriptor: [Erythrocyte Indices] explode all trees

#5: (erythrocyte distribution width):ti,ab,kw OR (RCDW):ti,ab,kw OR (RDW):ti,ab,kw OR (RDW-CV):ti,ab,kw OR (RDW-SD):ti,ab,kw OR (red blood cell distribution width):ti,ab,kw OR (Red Cell Indexes):ti,ab,kw OR (Erythrocyte Index):ti,ab,kw OR (Erythrocyte Indexes):ti,ab,kw OR (Red Cell Index):ti,ab,kw OR (Red Cell Indices):ti,ab,kw OR (Erythrocyte Size Determination):ti,ab,kw OR (Erythrocyte Size Determinations):ti,ab,kw OR (Mean Corpuscular Volume):ti,ab,kw OR (Mean Corpuscular Volumes):ti,ab,kw OR (Mean Cell Volume):ti,ab,kw OR (Mean Cell Volumes):ti,ab,kw OR (Mean Corpuscular Hemoglobin):ti,ab,kw OR (Mean Corpuscular Hemoglobins):ti,ab,kw OR (Erythrocyte Thickness):ti,ab,kw OR (Erythrocyte Diameter):ti,ab,kw OR (Erythrocyte Diameters):ti,ab,kw OR (Mean Corpuscular Hemoglobulin Concentration):ti,ab,kw OR (Mean Cell Hemoglobin Concentration):ti,ab,kw OR (Red Cell Distribution Width):ti,ab,kw OR (erythrocyte indices):ti,ab,kw

#6: #4 OR #5

#7: #3 AND #6

**SIRI:**

#1：MeSH descriptor: [Diabetic Nephropathies] explode all trees

#2：(Diabetic Kidney Disease):ti,ab,kw OR (Diabetic Kidney Diseases):ti,ab,kw OR (Diabetic Nephropathy):ti,ab,kw OR (Diabetic Glomerulosclerosis):ti,ab,kw OR (Intracapillary Glomerulosclerosis):ti,ab,kw OR (Kimmelstiel Wilson Disease):ti,ab,kw OR (Nodular Glomerulosclerosis):ti,ab,kw OR (Kimmelstiel Wilson Syndrome):ti,ab,kw OR (diabetes nephropathy):ti,ab,kw OR (diabetic kidney disease):ti,ab,kw OR (diabetic nephropathies):ti,ab,kw OR (diabetic renal disease):ti,ab,kw

#3：#1 OR #2

#4: (systemic inflammation reaction index):ti,ab,kw OR (systemic inflammation response index):ti,ab,kw OR (systemic inflammatory reaction index):ti,ab,kw OR (Systemic Inflammatory Response Index):ti,ab,kw OR (SIRI):ti,ab,kw

#5: #3 AND #4

**WOS: 521 records**

**SII:**

#1: TS=('diabetes nephropathy' OR 'diabetic kidney disease' OR 'diabetic nephropathies' OR 'diabetic renal disease' OR 'diabetic nephropathy' OR 'Diabetic Kidney Disease' OR 'Diabetic Kidney Diseases' OR 'Diabetic Glomerulosclerosis' OR 'Intracapillary Glomerulosclerosis' OR 'Kimmelstiel Wilson Disease' OR 'Nodular Glomerulosclerosis' OR 'Kimmelstiel Wilson Syndrom')

#2: TS=('systemic immune-inflammation index' OR 'systemic immune-inflammatory index' OR 'Systemic Immunity-inflammation Index' OR 'systemic immune inflammation index' OR 'systemic immune inflammatory index' OR 'SII' OR 'neutrophil × platelets/ lymphocyte')

#3: #1 AND #2

**PLR:**

#1: TS=('diabetes nephropathy' OR 'diabetic kidney disease' OR 'diabetic nephropathies' OR 'diabetic renal disease' OR 'diabetic nephropathy' OR 'Diabetic Kidney Disease' OR 'Diabetic Kidney Diseases' OR 'Diabetic Glomerulosclerosis' OR 'Intracapillary Glomerulosclerosis' OR 'Kimmelstiel Wilson Disease' OR 'Nodular Glomerulosclerosis' OR 'Kimmelstiel Wilson Syndrom')

#2: TS=('platelet to lymphocyte ratio' OR 'platelet/lymphocyte ratio' OR 'PLR' OR 'thrombocyte lymphocyte ratio' OR 'platelet lymphocyte ratio' OR 'platelet/lymphocyte')

#3: #1 AND #2

**MLR:**

#1: TS=('diabetes nephropathy' OR 'diabetic kidney disease' OR 'diabetic nephropathies' OR 'diabetic renal disease' OR 'diabetic nephropathy' OR 'Diabetic Kidney Disease' OR 'Diabetic Kidney Diseases' OR 'Diabetic Glomerulosclerosis' OR 'Intracapillary Glomerulosclerosis' OR 'Kimmelstiel Wilson Disease' OR 'Nodular Glomerulosclerosis' OR 'Kimmelstiel Wilson Syndrom')

#2: TS=('monocyte to lymphocyte ratio' OR 'monocyte/lymphocyte ratio' OR 'monocyte lymphocyte ratio' OR 'lymphocyte to monocyte ratio' OR 'lymphocyte monocyte ratio' OR 'lymphocyte/monocyte ratio' OR 'MLR' OR 'LMR')

#3: #1 AND #2

**MPV:**

#1: TS=('diabetes nephropathy' OR 'diabetic kidney disease' OR 'diabetic nephropathies' OR 'diabetic renal disease' OR 'diabetic nephropathy' OR 'Diabetic Kidney Disease' OR 'Diabetic Kidney Diseases' OR 'Diabetic Glomerulosclerosis' OR 'Intracapillary Glomerulosclerosis' OR 'Kimmelstiel Wilson Disease' OR 'Nodular Glomerulosclerosis' OR 'Kimmelstiel Wilson Syndrom')

#2: TS=('average platelet volume' OR 'average thrombocyte volume' OR 'mean thrombocyte volume' OR 'mean platelet volume' OR 'Mean Platelet Volumes' OR 'MPV')

#3: #1 AND #2

**RDW:**

#1: TS=('diabetes nephropathy' OR 'diabetic kidney disease' OR 'diabetic nephropathies' OR 'diabetic renal disease' OR 'diabetic nephropathy' OR 'Diabetic Kidney Disease' OR 'Diabetic Kidney Diseases' OR 'Diabetic Glomerulosclerosis' OR 'Intracapillary Glomerulosclerosis' OR 'Kimmelstiel Wilson Disease' OR 'Nodular Glomerulosclerosis' OR 'Kimmelstiel Wilson Syndrom')

#2: TS=('erythrocyte distribution width' OR 'RCDW' OR 'RDW' OR 'RDW-CV' OR 'RDW-SD' OR 'red blood cell distribution width' OR 'Red Cell Indexes' OR 'Erythrocyte Index' OR 'Erythrocyte Indexes' OR 'Red Cell Index' OR 'Red Cell Indices' OR 'Erythrocyte Size Determination' OR 'Erythrocyte Size Determinations' OR 'Mean Corpuscular Volume' OR 'Mean Corpuscular Volumes' OR 'Mean Cell Volume' OR 'Mean Cell Volumes' OR 'Mean Corpuscular Hemoglobin' OR 'Mean Corpuscular Hemoglobins' OR 'Erythrocyte Thickness' OR 'Erythrocyte Diameter' OR 'Erythrocyte Diameters' OR 'Mean Corpuscular Hemoglobulin Concentration' OR 'Mean Cell Hemoglobin Concentration' OR 'Red Cell Distribution Width' OR 'erythrocyte indices')

#3: #1 AND #2

**SIRI:**

#1: TS=('diabetes nephropathy' OR 'diabetic kidney disease' OR 'diabetic nephropathies' OR 'diabetic renal disease' OR 'diabetic nephropathy' OR 'Diabetic Kidney Disease' OR 'Diabetic Kidney Diseases' OR 'Diabetic Glomerulosclerosis' OR 'Intracapillary Glomerulosclerosis' OR 'Kimmelstiel Wilson Disease' OR 'Nodular Glomerulosclerosis' OR 'Kimmelstiel Wilson Syndrom')

#2: TS=('systemic inflammation reaction index' OR 'systemic inflammation response index' OR 'systemic inflammatory reaction index' OR 'Systemic Inflammatory Response Index' OR 'SIRI')

#3: #1 AND #2

**CNKI: 231篇**

**RDW:**

(主题：糖尿病肾病 + 糖尿病性肾病 + 糖尿病性肾小球硬化症 + 毛细管间性肾小球硬化症 + 结节性肾小球硬化症 + 糖尿病肾疾病）AND（主题：红细胞分布宽度 + 红细胞指数）

**PLR:**

（主题：糖尿病肾病 + 糖尿病性肾病 + 糖尿病性肾小球硬化症 + 毛细管间性肾小球硬化症 + 结节性肾小球硬化症 + 糖尿病肾疾病）AND（主题：血小板与淋巴细胞比值 + 血小板淋巴细胞比值 + PLR）

**MLR:**

（主题：糖尿病肾病 + 糖尿病性肾病 + 糖尿病性肾小球硬化症 + 毛细管间性肾小球硬化症 + 结节性肾小球硬化症 + 糖尿病肾疾病）AND（主题：单核细胞与淋巴细胞比值 + 单核细胞淋巴细胞比值 + MLR + LMR）

**MPV:**

（主题：糖尿病肾病 + 糖尿病性肾病 + 糖尿病性肾小球硬化症 + 毛细管间性肾小球硬化症 + 结节性肾小球硬化症 + 糖尿病肾疾病）AND（主题：平均血小板体积 + 血小板平均容积 + 血小板平均体积 + 平均血小板容积 + MPV）

**SII:**

（主题：糖尿病肾病 + 糖尿病性肾病 + 糖尿病性肾小球硬化症 + 毛细管间性肾小球硬化症 + 结节性肾小球硬化症 + 糖尿病肾疾病）AND（主题：系统性免疫炎症指数 + SII）

**SIRI:**

（主题：糖尿病肾病 + 糖尿病性肾病 + 糖尿病性肾小球硬化症 + 毛细管间性肾小球硬化症 + 结节性肾小球硬化症 + 糖尿病肾疾病）AND（主题：全系统性炎症反应指数 + SIRI）

**Wanfang: 339篇**

**RDW:**

主题: (红细胞分布宽度 OR 红细胞指数) and 主题:(糖尿病肾病 OR 糖尿病性肾病 OR 糖尿病性肾小球硬化症 OR 毛细管间性肾小球硬化症 OR 结节性肾小球硬化症 OR 糖尿病肾疾病)

**PLR:**

主题: (血小板与淋巴细胞比值 OR 血小板淋巴细胞比值 OR PLR) and 主题:(糖尿病肾病 OR 糖尿病性肾病 OR 糖尿病性肾小球硬化症 OR 毛细管间性肾小球硬化症 OR 结节性肾小球硬化症 OR 糖尿病肾疾病)

**MLR:**

主题: (糖尿病肾病 OR 糖尿病性肾病 OR 糖尿病性肾小球硬化症 OR 毛细管间性肾小球硬化症 OR 结节性肾小球硬化症 OR 糖尿病肾疾病) and 主题:(单核细胞与淋巴细胞比值 OR 单核细胞淋巴细胞比值 OR MLR OR LMR)

**MPV:**

主题: (平均血小板体积 OR 血小板平均容积 OR 血小板平均体积 OR 平均血小板容积 OR MPV) and 主题:(糖尿病肾病 OR 糖尿病性肾病 OR 糖尿病性肾小球硬化症 OR 毛细管间性肾小球硬化症 OR 结节性肾小球硬化症 OR 糖尿病肾疾病)

**SII:**

主题: (系统性免疫炎症指数 OR SII) and 主题:(糖尿病肾病 OR 糖尿病性肾病 OR 糖尿病性肾小球硬化症 OR 毛细管间性肾小球硬化症 OR 结节性肾小球硬化症 OR 糖尿病肾疾病)

**SIRI:**

主题: (全系统性炎症反应指数 OR SIRI) and 主题:(糖尿病肾病 OR 糖尿病性肾病 OR 糖尿病性肾小球硬化症 OR 毛细管间性肾小球硬化症 OR 结节性肾小球硬化症 OR 糖尿病肾疾病）

**VIP: 934篇**

**MLR:**

((((任意字段=单核细胞与淋巴细胞比值 OR 任意字段=单核细胞淋巴细胞比值) OR 任意字段=MLR) OR 任意字段=LMR) AND (((((任意字段=糖尿病肾病 OR 任意字段=糖尿病性肾病) OR 任意字段=糖尿病性肾小球硬化症) OR 任意字段=毛细管间性肾小球硬化症) OR 任意字段=结节性肾小球硬化症) OR 任意字段=糖尿病肾疾病))

**MPV:**

[(((((任意字段=平均血小板体积 OR 任意字段=血小板平均容积) OR 任意字段=血小板平均体积) OR 任意字段=平均血小板容积) OR 任意字段=MPV) AND (((((任意字段=糖尿病肾病 OR 任意字段=糖尿病性肾病) OR 任意字段=糖尿病性肾小球硬化症) OR 任意字段=毛细管间性肾小球硬化症) OR 任意字段=结节性肾小球硬化症) OR 任意字段=糖尿病肾疾病))](https://qikan.cqvip.com/Qikan/search/index?LngMySearHistoryIdGuid=5056704f-97a0-4778-8251-df8276daa418&from=Qikan_Article_History)

**RDW:**

[((任意字段=红细胞分布宽度 OR 任意字段=红细胞指数) AND (((((任意字段=糖尿病肾病 OR 任意字段=糖尿病性肾病) OR 任意字段=糖尿病性肾小球硬化症) OR 任意字段=毛细管间性肾小球硬化症) OR 任意字段=结节性肾小球硬化症) OR 任意字段=糖尿病肾疾病))](https://qikan.cqvip.com/Qikan/search/index?LngMySearHistoryIdGuid=5366d314-0355-4807-b0cc-0b5bcec06537&from=Qikan_Article_History)

**SII:**

[((((((任意字段=糖尿病肾病 OR 任意字段=糖尿病性肾病) OR 任意字段=糖尿病性肾小球硬化症) OR 任意字段=毛细管间性肾小球硬化症) OR 任意字段=结节性肾小球硬化症) OR 任意字段=糖尿病肾疾病) AND (任意字段=系统性免疫炎症指数 OR 任意字段=SII))](https://ycfw.hbmu.edu.cn:9000/http/1O8TRWQAIE15B007vrl5mZ5PL0Groiss93QW1/Qikan/search/index?LngMySearHistoryIdGuid=6393ac9b-9919-4aac-afff-5cfab07ace1b&from=Qikan_Article_History)

**SIRI:**

[((((((任意字段=糖尿病肾病 OR 任意字段=糖尿病性肾病) OR 任意字段=糖尿病性肾小球硬化症) OR 任意字段=毛细管间性肾小球硬化症) OR 任意字段=结节性肾小球硬化症) OR 任意字段=糖尿病肾疾病) AND (任意字段=全系统性炎症反应指数 OR 任意字段=SIRI))](https://qikan.cqvip.com/Qikan/search/index?LngMySearHistoryIdGuid=e3299b38-dde6-4122-9171-71c2a9051b91&from=Qikan_Article_History)

**CBM: 186篇**

**SII:**

#1: "糖尿病肾病"[不加权:扩展]

#2: "毛细管间性肾小球硬化症"[常用字段:智能] OR "糖尿病性肾小球硬化症"[常用字段:智能] OR "结节性肾小球硬化症"[常用字段:智能] OR "糖尿病肾疾病"[常用字段:智能] OR "糖尿病肾病"[常用字段:智能]

#3: (#2) OR (#1)

#4: "系统性免疫炎症指数"[常用字段:智能] OR "SII"[常用字段:智能]

#5: (#4) AND (#3)

**PLR:**

#1: "糖尿病肾病"[不加权:扩展]

#2: "毛细管间性肾小球硬化症"[常用字段:智能] OR "糖尿病性肾小球硬化症"[常用字段:智能] OR "结节性肾小球硬化症"[常用字段:智能] OR "糖尿病肾疾病"[常用字段:智能] OR "糖尿病肾病"[常用字段:智能]

#3: (#2) OR (#1)

#4: "血小板与淋巴细胞比值"[常用字段:智能] OR "血小板淋巴细胞比值"[常用字段:智能] OR "PLR"[常用字段:智能]

#5: (#4) AND (#3)

**MLR:**

#1: "糖尿病肾病"[不加权:扩展]

#2: "毛细管间性肾小球硬化症"[常用字段:智能] OR "糖尿病性肾小球硬化症"[常用字段:智能] OR "结节性肾小球硬化症"[常用字段:智能] OR "糖尿病肾疾病"[常用字段:智能] OR "糖尿病肾病"[常用字段:智能]

#3: (#2) OR (#1)

#4: "单核细胞与淋巴细胞比值"[常用字段:智能] OR "单核细胞淋巴细胞比值"[常用字段:智能] OR "MLR"[常用字段:智能] OR "LMR"[常用字段:智能]

#5: (#4) AND (#3)

**MPV:**

#1: "糖尿病肾病"[不加权:扩展]

#2: "毛细管间性肾小球硬化症"[常用字段:智能] OR "糖尿病性肾小球硬化症"[常用字段:智能] OR "结节性肾小球硬化症"[常用字段:智能] OR "糖尿病肾疾病"[常用字段:智能] OR "糖尿病肾病"[常用字段:智能]

#3: (#2) OR (#1)

#4: "平均血小板体积"[不加权:扩展]

#5: "平均血小板体积"[常用字段:智能] OR "血小板平均容积"[常用字段:智能] OR "血小板平均体积"[常用字段:智能] OR "平均血小板容积"[常用字段:智能] OR "MPV"[常用字段:智能]

#6: (#5) OR (#4)

#7: (#6) AND (#3)

**RDW:**

#1: "糖尿病肾病"[不加权:扩展]

#2: "毛细管间性肾小球硬化症"[常用字段:智能] OR "糖尿病性肾小球硬化症"[常用字段:智能] OR "结节性肾小球硬化症"[常用字段:智能] OR "糖尿病肾疾病"[常用字段:智能] OR "糖尿病肾病"[常用字段:智能]

#3: (#2) OR (#1)

#4: "红细胞指数"[不加权:扩展]

#5: "红细胞分布宽度"[常用字段:智能] OR "红细胞指数"[常用字段:智能]

#6: (#5) OR (#4)

#7: (#6) AND (#3)

**SIRI:**

#1: "糖尿病肾病"[不加权:扩展]

#2: "毛细管间性肾小球硬化症"[常用字段:智能] OR "糖尿病性肾小球硬化症"[常用字段:智能] OR "结节性肾小球硬化症"[常用字段:智能] OR "糖尿病肾疾病"[常用字段:智能] OR "糖尿病肾病"[常用字段:智能]

#3: (#2) OR (#1)

#4: "全系统性炎症反应指数"[常用字段:智能] OR "SIRI"[常用字段:智能]

#5: (#4) AND (#3)
